# Modulation of the Activity and Regioselectivity of a Glycosidase: Development of a Convenient Tool for the Synthesis of Specific Disaccharides

**DOI:** 10.3390/molecules26185445

**Published:** 2021-09-07

**Authors:** Yari Cabezas-Pérusse, Franck Daligault, Vincent Ferrières, Olivier Tasseau, Sylvain Tranchimand

**Affiliations:** 1 Ecole Nationale Supérieure de Chimie de Rennes, CNRS, ISCR (Institut des Sciences Chimiques de Rennes)—UMR 6226, Université de Rennes, F-35000 Rennes, France; cabezas.yari@gmail.com (Y.C.-P.); vincent.ferrieres@ensc-rennes.fr (V.F.); olivier.tasseau.1@ensc-rennes.fr (O.T.); 2 CNRS, UFIP (Unité de Fonctionnalité et Ingénierie des Protéines)—UMR 6286, Université de Nantes, F-44000 Nantes, France; franck.daligault@univ-nantes.fr

**Keywords:** galactofuranoside, transglycosylation, molecular dynamics, mutagenesis

## Abstract

The synthesis of disaccharides, particularly those containing hexofuranoside rings, requires a large number of steps by classical chemical means. The use of glycosidases can be an alternative to limit the number of steps, as they catalyze the formation of controlled glycosidic bonds starting from simple and easy to access building blocks; the main drawbacks are the yields, due to the balance between the hydrolysis and transglycosylation of these enzymes, and the enzyme-dependent regioselectivity. To improve the yield of the synthesis of β-d-galactofuranosyl-(1→X)-d-mannopyranosides catalyzed by an arabinofuranosidase, in this study we developed a strategy to mutate, then screen the catalyst, followed by a tailored molecular modeling methodology to rationalize the effects of the identified mutations. Two mutants with a 2.3 to 3.8-fold increase in transglycosylation yield were obtained, and in addition their accumulated regioisomer kinetic profiles were very different from the wild-type enzyme. Those differences were studied in silico by docking and molecular dynamics, and the methodology revealed a good predictive quality in regards with the regioisomer profiles, which is in good agreement with the experimental transglycosylation kinetics. So, by engineering *Ct*Ara*f*51, new biocatalysts were enabled to obtain the attractive central motif from the *Leishmania* lipophosphoglycan core with a higher yield and regioselectivity.

## 1. Introduction

**Figure 1:**
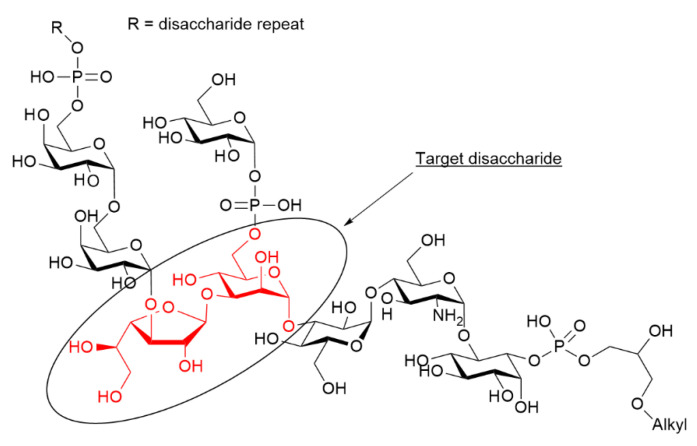
LPG general structure with a special emphasis on the core heptasaccharide and the target disaccharide.

Leishmaniases are neglected tropical diseases occurring in several intertropical areas in South America, Africa and Asia. More than 1 million people are infected by these parasites every year, with various consequences, as the clinical forms of the disease can be either cutaneous or visceral [1]. In the first case, the disease can heal spontaneously and lead to disgraceful scars. The second case is much more preoccupying, because symptoms are very difficult to distinguish and without treatment, overinfections are almost inevitably occurring, causing death in the medium term [1]. The actual treatments are very expensive and necessitate heavy instruments in medical facilities of countries that cannot always afford them. There is an urgent need to develop new strategies for the treatment and the diagnosis of these diseases. An interesting field of investigation is the lipophosphoglycans (LPGs) present at the surface of *Leishmania* cells [2]. LPGs are virulence factors constituted by 4 distinct domains: a lipid anchor, a core heptasaccharide, a polymeric repetitive tail and a cap. The core heptasaccharide is of particular interest for two reasons: it constitutes a retained feature among *Leishmania* species and it contains a galactofuranose (Figure 1), a rare carbohydrate unit that is xenobiotic to mammals [3], conferring a keen relevance to this moiety for diagnosis and treatment development [4]. The core heptasaccharide was already been obtained by chemical synthesis, but this was a very long and difficult multistep process, with a large number of protection and deprotection steps inherent to oligosaccharide synthesis. The presence of a galactofuranose moiety was an additional difficulty in this synthesis, as it is not the thermodynamically favored form of galactose and so implies particular conditions to be maintained [5,6]. Due to the complexity of its synthesis, this core heptasaccharide has not yet been evaluated for medical applications. In order to access more easily such kind of galactofuranoconjugates, with a limited number of steps and in a way that follows the concepts of green chemistry, our team developed tools to obtain galactofuranoconjugates through a biocatalytic process involving the use of an arabinofuranosidase from *Clostridium thermocellum* (*Ct*Ara*f*51) [7,8,9]. In the present study we engineered this enzyme to obtain the disaccharide β-d-galactofuranosyl-(1→3)-d-mannopyranose, the central part of this core heptasaccharide, with a higher regioselectivity and a higher yield. With the aim of following the green chemistry rules, based on our previous results, we replaced the generally used *p*-nitrophenyl derivatives as donor substrates by a fully biosourced donor, *n*-octyl β-d-galactofuranoside [7].

## 2. Results and Discussion

### 2.1. Transglycosylation Profiles with p-Nitrophenyl α-d-Mannopyranoside (Manp-pNP) as Acceptor Using the Wild-Type (WT) CtAraf51 Enzyme

**Scheme 1:**
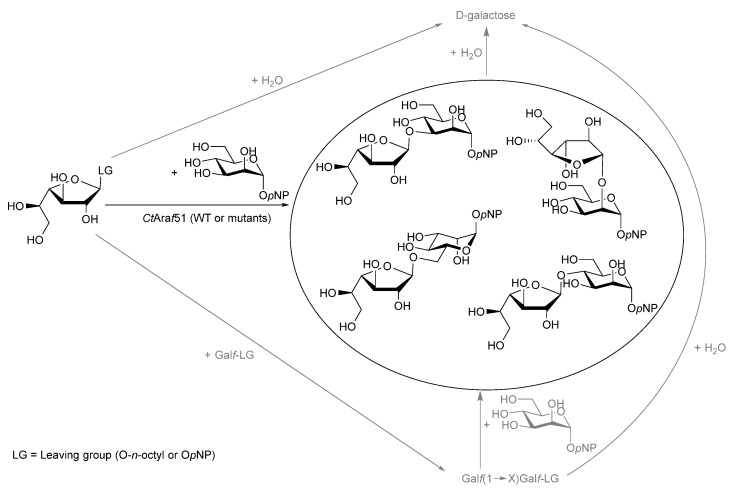
Transglycosylation between a β-galactofuranosyl donor and an α-mannopyranosyl acceptor, and the side reactions occurring during the catalysis.

*Ct*Ara*f*51 is a thermostable arabinofuranosidase from the GH51 family that was shown to be an interesting tool for the synthesis of galactofuranosides [7,8,9], with various efficiencies depending on the acceptor substrates. The enzyme follows the typical retaining glycosidase mechanism, i.e., a double-displacement mechanism. First, the nucleophilic residue (E292) attacks the anomeric carbon of a donor substrate leading, with the concomitant help of an acid/base residue (E173), to the formation of a covalent glycosyl-enzyme intermediate and the release of the aglycone part of the donor. Then, a nucleophilic acceptor (water, alcohol) is activated by the later acid/base residue and attacks back the glycosyl-enzyme intermediate to release either hydrolysis or transglycosylation products. In transglycosylation reactions, the main limitation is the competition between the different reactions catalyzed by the glycosidase occurring in parallel during the time course of the process (Scheme 1). To optimize the production of a desired target by such means, kinetic monitoring is necessary to prevent subsequent hydrolysis of the target.

The target disaccharide is the *p*-nitrophenyl β-d-galactofuranosyl-(1→3)-α-d-mannopyranoside (G(1→3)M). The first step in the development of a biocatalytic methodology to synthesize this compound was to set the optimized operating conditions with the wild-type enzyme (WT). Due to the competition between autocondensation and transglycosylation reactions during the process (Scheme 1), *n*-octyl β-d-galactofuranoside (Gal*f*-octyl) was chosen as the donor substrate instead of its commonly used *p*NP counterpart, and Man*p*-*p*NP as the acceptor. With these substrates, the obtained *n*-octyl oligosaccharides (autocondensation products) were easily separated from the *p*-nitrophenyl oligosaccharides (transglycosylation products), the octyl donor was suitable for such reactions as it was already observed [7].

To favor transglycosylation reactions over hydrolysis, high substrate concentrations were intended. As both Gal*f*-octyl and Man*p*-*p*NP have relatively low solubilities in aqueous buffers at room temperature, the temperature was set to 60 °C: solubilities of both substrates increased dramatically (up to 10 mM for Gal*f*-octyl and 100 mM for Man*p*-*p*NP) under this condition and the enzyme remained stable and active for several hours [10]. The donor concentration was then established at 10 mM and the donor/acceptor ratios from 1/2 to 1/10 were evaluated. 

Beyond the 1/6 ratio, no significant differences in the transglycosylation product accumulations were observed (Supplementary Materials Figure S1), this ratio was then kept for all the following experiments. Finally, the enzyme concentration was adjusted to reach the maximal concentration of the total tranglycosylation products within 8 h: 0.5 U/10 µmol of donor substrate were required.

**Figure 2:**
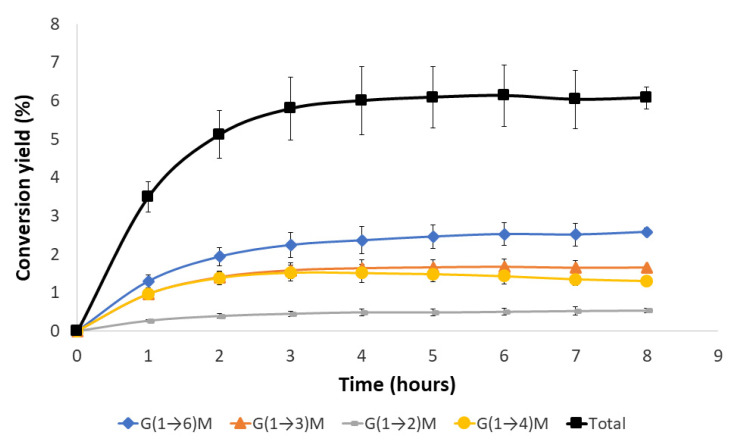
Transglycosylation kinetics between Gal*f*-octyl (10 mM) and Man*p*-*p*NP (60 mM) catalyzed by *Ct*Ara*f*51 WT at 60 °C. The error bars represent the standard deviation of three independent experiments.

The conversion kinetics obtained in these optimized conditions with the *Ct*Ara*f*51 WT are presented in Figure 2. A plateau after 3 h was reached for the studied disaccharides. The conversion into G(1→X)M transglycosylation products remained very low, with a total amount of heterodisaccharides around only 6%, the main regioisomer accumulated was the (1→6) product instead of the desired (1→3). Beyond 8 h, the total amount of transglycosylation products slowly decreased due to secondary hydrolysis.

### 2.2. Mutagenesis Strategy

**Figure 3:**
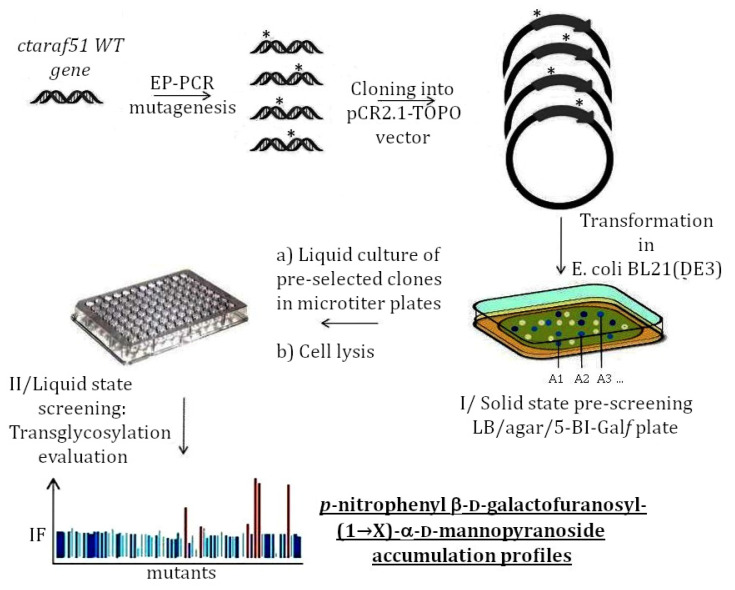
Mutagenesis strategy of the arabinofuranosidase *Ct*Araf51. IF: improvement factor, calculated according to Equation (1). The * outline the random mutations in the gene sequence.

In order to improve the transglycosylation efficiency and regioselectivity towards the G(1→3)M regioisomer, a random mutagenesis strategy was applied. Indeed, unlike other glycosidases [11] no water channel was recognizable to rationalize the modification of the hydrolysis/transglycosylation balance. The overall strategy is shown in Figure 3: a library of variants was generated by error-prone PCR and screened for transglycosylation ability. The screening consisted of a two steps process according to Koné et al. [12] to select variants showing improved transglycosylation/hydrolysis ratio (selection of low hydrolytic variants in the sole presence of the donor in the first step, and of better transglycosylation variants in presence of acceptor in the second step). However, in our case the second step was carried out in liquid phase with crude extracts because Man*p*-*p*NP cannot go through the cell membrane. In the first step, the EP-PCR library was transformed in *E. coli*, spread on a nitrocellulose membrane and grown over a LB-agar-ampicillin plates. After overnight growth, the membrane was transferred to a new agar plate containing buffer, ampicillin, IPTG and 5-bromoindolyl β-d-galactopyranoside (5-BI-Gal*f*). The less hydrolytic pale blue colonies were selected (120 isolated colonies called MYC 1 to 120) and the screening with and without the acceptor was performed in the solution to highlight the best transglycosylation variants.

Transglycosylation reactions with Gal*f*-octyl and Man*p*-*p*NP were conducted in the optimized conditions with the crude extracts as enzyme solutions. As a control, the WT enzyme was produced in the exact same conditions. The transglycosylation kinetics were monitored by HPLC after 3 h and 17 h of reaction for each individual mutant and compared with the profiles of the WT. As the amount of the four regioisomers was very small, their sum was used instead of their individual concentrations during the screening. Under these conditions, with the WT enzyme, the overall quantity of G(1→X)M disaccharides was higher at t = 3 h than 17 h due to secondary hydrolysis. The sum obtained at t = 3 h was then used as a reference to screen mutants with an overall increase in transglycosylation products accumulation. In addition to the time points after 3 h, the time points at 17 h were also analyzed for every mutant to consider their potentially slower activity resulting from an altered hydrolysis/transglycosylation balance. An improvement factor (IF) was calculated according to Equation (1):

IF = ΣA^Mut^_trans(tmax)_/ΣA^WT^_trans(3h)_, (1)

where ΣA^Mut^_trans(tmax)_ is the highest sum of the areas of peaks corresponding to G(1→X)M disaccharides for the considered mutant and ΣA^WT^_trans(3h)_ is the sum of the areas of pics corresponding to G(1→X)M disaccharides obtained with *Ct*Ara*f*51 WT at t = 3h. The IFs of the selected mutants are presented in the Supplementary Materials (Figure S2).

Only a few mutants showed an improved transglycosylation product accumulation compared with the WT (IF > 1) in the screening conditions, most of them having as expected a lower overall activity resulting in a delayed maximum conversion yield (ΣA^Mut^_trans(tmax)_ at t = 17 h instead of 3 h for the WT). From this screening batch, only five mutants showed an IF > 2 and were further characterized. Plasmids from these mutants were isolated and sequenced. The observed mutations are reported in Table 1. In fact, three of them were identical (MYC69, MYC70 and MYC98).

### 2.3. Transglycosylation Kinetics with the Selected Mutants

**Figure 4:**
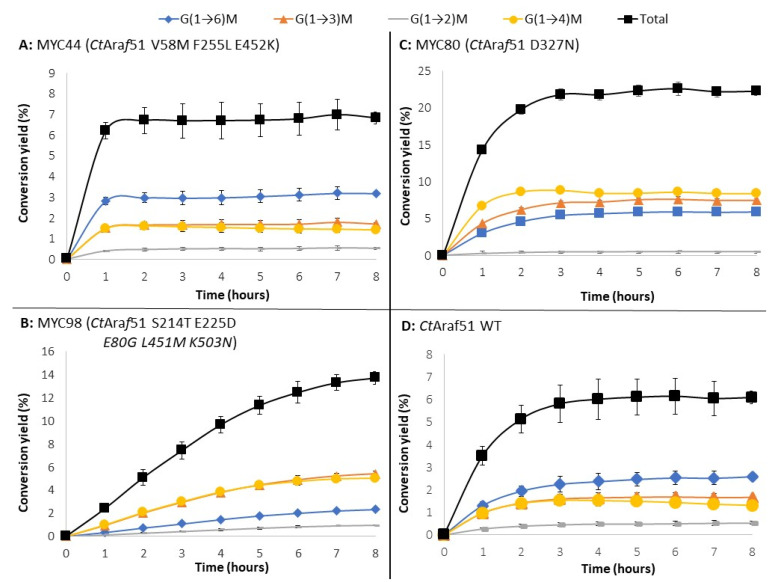
Transglycosylation kinetics with the *Ct*Ara*f*51 WT enzyme and mutants directly selected during the screening ((**A**): MYC44, (**B**): MYC98, (**C**): MYC80, (**D**): WT). Mutations labelled in italic have no influence either on the transglycosylation yield nor on the regioselectivity. A total of 10 mM of Gal*f*-octyl and 60 mM of Man*p*-*p*NP were used at 60 °C. The error bars represent the standard deviation of three independent experiments.

Transglycosylation kinetics were monitored in the optimized conditions to compare more accurately the regioisomer profiles and the conversion maxima. The kinetics are depicted in Figure 4. Despite the standardization of the activity units, differences in the required time to reach the plateau were observed. While difficult to anticipate, these differences were expected: indeed, the activity units only depict the primary hydrolysis and autocondensation of the donor in the absence of external acceptor whereas in transglycosylation conditions, much more reactions occur in parallel (Scheme 1). The mutants are most likely impaired in the balance between these different reactions. 

The MYC44 mutant showed similar profiles (regioisomers and yields) to the WT, suggesting an artifact of the increased IF observed during the screening, and was discarded. Then, on the contrary the two other mutants had very interesting kinetic profiles: they both showed a significant increase in the overall yield of transglycosylation (2.3 and 3.8-fold increase for MYC98 and MYC80, respectively), but also the proportions of the different regioisomers were altered (Figure 4B,C). Indeed, the major accumulated disaccharide was the G(1→6)M with the *Ct*Ara*f*51 WT (around 40% of the total amount of transglycosylation disaccharides), its proportion being reduced to 26% with MYC80 and even 17% with MYC98. In both cases, instead of G(1→6)M as the major product, an almost equal proportion of G(1→3)M and G(1→4)M was accumulated as the main products.

**Figure 5:**
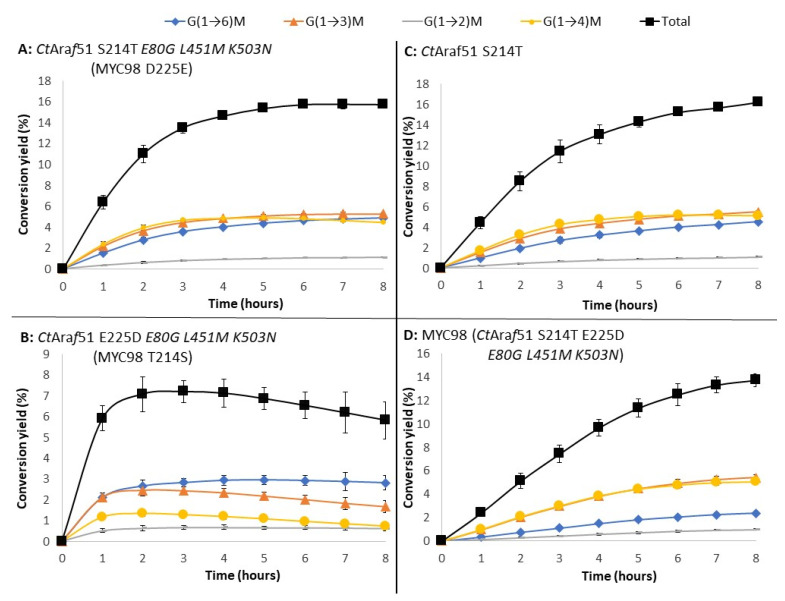
Transglycosylation kinetics with the mutant MYC98 and its derived mutants ((**A**): MYC98 D225E, (**B**): MYC98 T214S, (**C**): *Ct*Ara*f*51 S214T, (**D**): MYC98). Mutations labelled in italic have no influence either on the transglycosylation yield nor on the regioselectivity. A total of 10 mM of Gal*f*-octyl and 60 mM of Man*p*-*p*NP were used at 60 °C. The error bars represent the standard deviation of three independent experiments.

As the mutant MYC98 bears 5 mutations, to investigate their roles in the modulation of the enzymatic activity, every mutation was reversed one at a time by point mutagenesis, and the profiles with the five new mutants (MYC98 G80E, MYC98 T214S, MYC98 D225E, MYC98 M451L, and MYC98 N503K) were evaluated under the same conditions (Figure 5). Mutations at positions 80, 451 and 503 appeared to have no effects on the enzymatic activity as the kinetic profiles obtained for the corresponding mutants (MYC98 G80E, MYC98 M451L and MYC98 N503K) were superimposable to the MYC98 profile (data not shown). Mutations at the two remaining positions had a very different influence on the enzymatic activity: the S214T mutation was the main mutation responsible for the increase in the overall transglycosylation yield while the E225D mutation was modifying the proportions of the resulting disaccharides. Indeed, as shown in Figure 5, with the mutant *Ct*Ara*f*51 E225D *E80G L451M K503N* (MYC98 T214S), the total amount of G(1→X)M disaccharides was reduced to 7%, i.e., almost the same yield as with the *Ct*Ara*f*51 WT, while it reached 16% with *Ct*Ara*f*51 S214T *E80G L451M K503N* (MYC98 D225E), an even higher yield than with MYC98. This later difference seems to be related exclusively to the G(1→6)M accumulation: G(1→4)M, G(1→3)M, and G(1→2)M were accumulated to the same level (5%, 5% and 1%, respectively), but G(1→6)M was reduced from 5 to 2% when there was the E225D mutation. This observation concerning the E225D mutation was also consistent with the profiles without the mutation at position 214 (between the *Ct*Ara*f*51 WT and *Ct*Ara*f*51 E225D *E80G L451M K503N* (MYC98 T214S)): at the points with the maximal conversion yield, the major product was by far G(1→6)M with the *Ct*Ara*f*51 WT and its proportion decreased to the level of the G(1→3)M with *Ct*Ara*f*51 E225D *E80G L451M K503N* (MYC98 T214S) (Figure 2 and Figure 5). With the later mutant it can also be observed that the accumulation of G(1→3)M and G(1→4)M was decreasing over time after 2 h, meaning this mutant displays a slightly higher secondary hydrolysis contributing to the poor overall transglycosylation products accumulation. To ensure that there was no hidden cross-effect between the MYC98 mutations, the single mutant *Ct*Ara*f*51 S214T was also generated and used in transglycosylation reaction (Figure 5). As expected, its kinetic profile was identical to the profile of *Ct*Ara*f*51 S214T *E80G L451M K503N* (MYC98 D225E), confirming the absence of influence from the mutations at positions 80, 451 and 503 (Figure 5A,C). 

### 2.4. Molecular Modelling

#### 2.4.1. Methodology and Molecular Dynamics (MD) on Glycosyl-Enzymes

**Figure 6:**
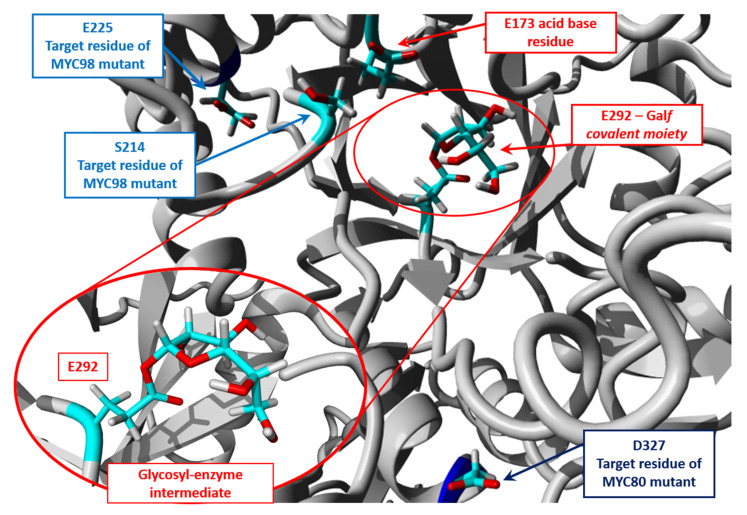
Molecular view of the wild-type Gal*f* covalent complex with highlight on MYC98 and MYC80 mutations and focus on the glycosidic moiety.

As modeling the different disaccharides in the enzyme would provide more insight about the secondary hydrolysis than their synthesis, a new strategy was adopted: modeling the acceptor substrate in interaction with the glycosyl-enzyme intermediate, to mimic closely the second half-reaction of the transglycosylation. In order to compare the motions leading to the different regioisomers rather than finding only the most suitable one, the acceptor was initially positioned in optimized ways considering the different (1→X) Man*p*-*p*NP approaches instead of a neutral position, and their evolutions over time were evaluated. These in silico studies on the acceptor binding site follow our previous MD investigations about the interactions of the Gal*f*-*p*NP donor with the catalytic pocket, in particular with hydrophobic residues [9]. Considering the very low yield for G(1→2)M disaccharide, its formation in the binding site of enzyme complexes and (1→2) orientation of the acceptor was not considered. So, simulations focused on the formations of the main disaccharides: G(1→3)M, G(1→4)M, G(1→6)M. For every disaccharide regioisomers, the initial ^4^C_1_ conformation was retained for the Man*p* ring. Since experimentally no modifications in activity were related to positions 80, 451 and 503, in silico mutagenesis were focused exclusively on S214, E225, and D327 residues (Figure 6). First, Gal*f*-enzyme covalent intermediates were studied by MD either with the wild type or the mutants as enzyme models. Only minor differences were observed between the *Ct*Ara*f*51 WT and the mutants.

#### 2.4.2. Simulations of the Acceptor Approaching Glycosyl-Enzymes

**Scheme 2:**
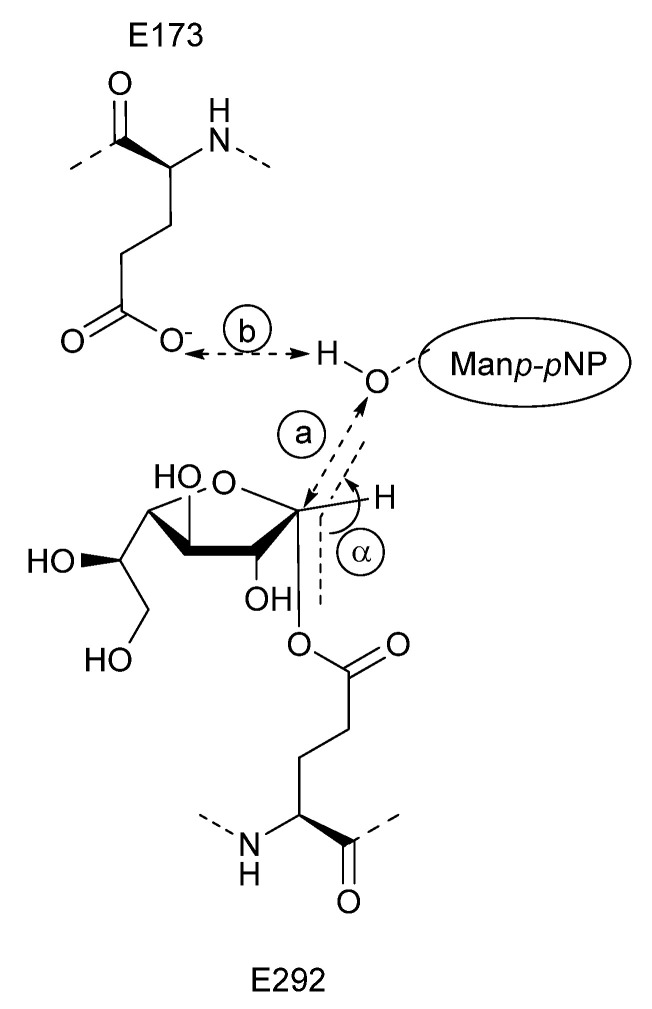
Distances (a_X_,b_X_) and angle α_X_ to study the Man*p*-*p*NP orientations approaching Gal*f*-enzyme intermediate.

From global docking, four complexes were selected to perform local docking for each G(1→X)M regioisomer (X = 3, 4, and 6) and one main conformation was found in each case. These docked complexes were superimposed with their corresponding MD last Gal*f*-enzyme complexes. The methodology was then to consider the reaction backward: the simulation starts with the Man*p* moiety perfectly oriented to allow the desired (1→X) glycosidic bond formation, and its evolution over time is evaluated. To do so, after the superimposition, only the Man*p*-*p*NP moiety was conserved from the docking of the disaccharide, and fused with the corresponding final MD of the Gal*f*-enzyme intermediate. For each of the orientations, new MD over 25 to 30 ns enabled the observations of the Man*p*-*p*NP binding evolutions, and complexes with the best binding energy were kept in each case. These complexes were compared with the starting conformations and between them, on the basis of the glycosidic bond formation distance (a_X_ = distance between the oxygen of the considered Man*p*-*p*NP hydroxyl and the anomeric carbon of the Gal*f* moiety), the distance between the acid-base residue and the hydroxyl to be activated (b_X_) and the angle Oε_E292_-C1-O_X,Man*p*_ (α_X_) (Scheme 2, Table 2, Supplementary Materials Table S1). Values higher than 109.5° for the α_X_ angle were expected to allow the nucleophilic attack leading to the glycosidic bond (i.e., the hydroxyl group above the plane formed by the cyclic oxygen of the Gal*f* moiety, Gal*f*-C_2_ and Gal*f*-H_anomeric_). In general, shorter distances a_X_ were between 3 and 4 Å.

With the *Ct*Ara*f*51 WT enzyme or all the evaluated mutants, for MD simulations starting with the Man*p* moiety in the (1→3) orientation toward the Gal*f*, the Man*p* kept close conformations favoring the (1→3) glycosylation. When Man*p* was initially in the (1→4) orientation, simulation remained in favor of the (1→4) orientation only with the D327N mutant. All the other simulations led to conformations where the distance a_3_ was comparable (*Ct*Ara*f*51 WT, S214T mutant, S214T E225D double mutant) or even much shorter than a_4_ (E225D single mutant). 

Within all the simulations with the *Ct*Ara*f*51 WT, the shorter obtained a_3_ and a_4_ distances were close to each other (5.60 and 6.74 Å respectively, i.e., less than a 17% difference), which is consistent with the experimental data as G(1→3)M and G(1→4)M were accumulated at comparable levels. The same observations can be performed with the S214T mutant, the D327N mutant, and the S214T E225D double mutant (MYC98). Comparisons of a_3_ and a_4_ shorter distances within the E225D mutant simulations are also consistent with the experimental data: a_4_ (8.12 Å) was significantly higher than a_3_ (5.37 Å, i.e., a 34% difference) and experimentally G(1→4)M accumulation was only half of G(1→3)M. 

Comparisons of the b_3_ and b_4_ distances (Supplementary Materials Table S1) are similar to those of the a_X_ distances: they are almost equal for the S214T mutant, the D327N mutant and the S214T E225D double mutant and in favor of the G(1→3)M formation for the E225D single mutant. This parameter b_X_ seems a bit more in favor of the G(1→3)M formation for the *Ct*Ara*f*51 WT, but might have a lower weight on the overall activity than the a_X_ distance. The α_X_ angles are more difficult to rationalize: they were always in favor of the (1→3) bond formation and even rather unfavorable for G(1→4)M formation in the cases of the S214T mutant and the S214T E225D double mutant despite the experimental data (Supplementary Materials Table S1 and Figure S3). As the furanose ring distortion during the catalytic step [13,14] was not considered during the simulations, these angles might nonetheless remain suitable for the nucleophilic attack to occur. 

**Figure 7:**
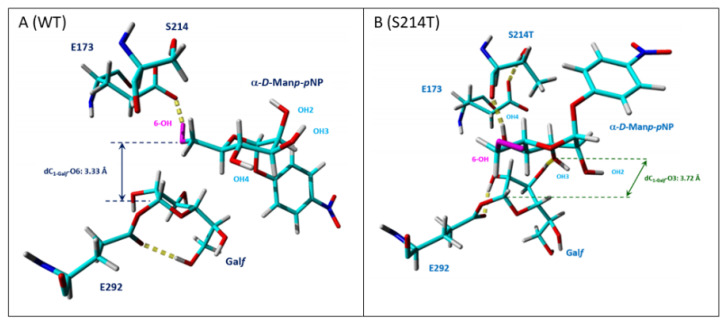
*Ct*Ara*f*51 WT (**A**)/*Ct*Ara*f*51 S214T (**B**)-Gal*f* complexes interacting with Man*p*-*p*NP after MD. Man*p*-*p*NP was initially (1→6) oriented. (Man*p*(6-OH) are highlighted in magenta; stackable complexes with a RMSD: 1.3303 Å on 7991 matched atoms).

Different behaviors were observed with MD simulations starting with (1→6) orientations of Man*p* (Figure 7, Supplementary Materials Figure S3). With the *Ct*Ara*f*51 WT, the Man*p* moiety remained in favor of G(1→6)M formation. With the single E225D mutant, a close result was obtained in favor of the (1→6) isomer, but the α_6_ angle was less favorable than the one obtained with the *Ct*Ara*f*51 WT. With the D327N mutant, the Man*p* moiety turned slightly in favor of the (1→4) orientation over the (1→6) (a_4_ = 3.30 Å, a_6_ = 4.10 Å). The results were very different with the S214T E225D double mutant: a translation and a progressive rotation of the acceptor (C_1_-C_1′_: 3.2 Å and torsion θ: O_endo_-C_1_-C_1′_-O_endo’_: 92.5°) disadvantaged any G(1→X)M formation (the shorter a_X_ value was a_6_ = 7.57 Å). Finally, during MD with the S214T mutant, the Man*p* moiety flipped to propose a preferential G(1→3)M orientation and an unfavorable G(1→6)M one. These results were again in good agreement with the experimental data: the single mutants S214T and D327N, and the S214T E225D double mutant (MYC98) had severely reduced proportions of the G(1→6)M regioisomer, compared with their (1→3) and (1→4) counterparts (Figure 4 and Figure 5).

These results corroborated the experimental data: the G(1→6)M isomer is the major one for the wild type and the E225D single mutant, confirmed in silico by smaller a_6_ and b_6_ distances (G(1→6)M proportion being lower for the E225D mutant due to a less favorable α_6_ angle). For the three other considered mutants, G(1→6)M accumulation was greatly reduced, and MD simulations showed indeed the instability of this orientation (Supplementary Materials Figure S3). G(1→4)M and G(1→3)M were accumulated at almost the same level with all the enzymes except for the E225D single mutant and once again *in silico*, comparable a_X_ and b_X_ values were obtained when simulating the formation of the two regioisomers except precisely for the mutant E225D where the hydroxyl at position 4 always remained further away from the Gal*f* moiety. 

In order to analyze further the modification leading to changes in regioselectivity, B-factor and RMSF calculations were conducted. Mutations S214T and D327N are associated with particular motions of β4α4 loop at +1 catalytic subsite [10] and β7α7 loop at −1 subsite (*Ct*Ara*f*51 secondary structures are summarized in Supplementary Materials Figure S4). The S214T mutation induces additional steric and hydrophobic hindrances leading to a change in the Man*p*-*p*NP interactions with W178 residue (hydrophobic and CH-π interactions). So, β4α4 conformational modifications can favor the opening of the catalytic site by rotating the W178 indole plane (20 to 25°), and benefit the G(1→3)M formation at the expense of the G(1→6)M (Supplementary Materials Figure S5A,C). As part of α7′ helix, D327N mutation appears to enhance flexibilities of neighboring secondary regions: α7α7′ loop, α7 helix and β7α7 loop. B-factor colorized maps and the thermal mobilities per residue indicate conformational motions from the α7α7′ loop. Notably, the hydrophobic pocket of the catalytic site with L318 and L319 residues [9] presented an increased B-factor, in particular with (1→3) and (1→6) orientations (Supplementary Materials Figure S6A,C). W296 residue (β7 strand, highly conserved (97%) in GH51 family) enabled conformational modifications by π-stacking and hydrophobic interactions with the acceptor and the other important conserved residue, Y244 (β6 strand) [13]. β7α7 loop’s flexibility appeared important in the interactions between *Ct*Ara*f*51 D327N-Gal*f* glycosyl-enzyme and Man*p*-*p*NP (Supplementary Materials Figure S3E). With the initial (1→3) orientation of the acceptor, compared with the *Ct*Ara*f*51 WT, the D327N mutant induced a rotation of Man*p* ring (O3_ManpA1_C_1-Galf_O3 _ManpA2_: 30°) resulting in increased π-stacking interactions between the *p*NP arm of the acceptor and the residues W296 and Y244. In comparison with the *Ct*Ara*f*51 WT with the D327N mutant, during the initial (1→6) Man*p*-*p*NP approach, the acceptor moved away from catalytic -1 subsite to interact only with +1 subsite resulting in the loss of π–π interactions between W296 and the *p*NP moiety of Man*p*-*p*NP, keeping distances a_X_ smaller for 4-OH than 6-OH. Unlike the initial (1→3) and (1→6) orientations, during simulations starting from the (1→4) orientation, a_4_ and b_4_ suggest a preferred disaccharide G(1→4)M formation (Table 2, Supplementary Materials Table S1 and Figure S3). B-factor analyses per residue for the *Ct*Ara*f*51 WT and the D327N mutant depicted similar thermal mobilities (Supplementary Materials Figure S6). Indeed, in this case, the β7α7 loop seems less involved as no significant interactions between the Man*p*-*p*NP and residues W296 and Y244 were depicted in both cases (Supplementary Materials Figure S3A,E), instead privileged interactions with the β5α5 loop were observed with the D327N mutant: the hydrogen bond with S214 and π-stacking between W178 indole side chain and *p*NP arm.

## 3. Materials and Methods

### 3.1. Materials

Solvents, reagents and other chemicals were purchased from Merck KGaA (Darmstadt, Germany) or Acros Organics (Geel, Belgium) and used without further purifications if not stated otherwise. The *p-*nitrophenyl α-d-mannopyranoside (Man*p*-*p*NP) and *p-*nitrophenyl β-d-galactofuranoside (Gal*f*-*p*NP) were purchased from Carbosynth Limited, UK. The *n-*octyl β-d-galactofuranoside (Gal*f*-octyl) was synthesized according to Ferrières et al. [15]. The donor substrate 5-bromo-indolyl-β-d-galactofuranoside (5-BI-Gal*f*) synthesis is described in Supplementary Materials SE1 (adapted from Berlin et al. [16]). The NMR spectra were recorded with a Brüker ARX 400 spectrometer (Billerica, MA, USA) at 400 MHz for ^1^H and 100 MHz for ^13^C. Chemical shifts are given in δ-units (ppm) measured from the solvent signal. Coupling constants J were calculated in Hertz (Hz). Abbreviations were used to precise signal multiplicity: singlet (s), doublet (d), triplet (t), multiplet (m), doublet doublet (dd), and apparent (app.). HRMS were measured at the Centre Régional de Mesures Physiques de l’Ouest (CRMPO, Université Rennes 1, Rennes, France) with a micrOTOF-Q II (Brüker, Billerica, MA, USA). Optical rotations were measured on a Perkin-Elmer 341 Polarimeter (PerkinElmer SAS, Villebon-sur-Yvette, France). Analytical HPLC were carried out using a Shimadzu LCMS-2020 Prominence UFLC system (Shimadzu France SAS, Marne la Vallée, France) with a Thermo Scientific Accucore C18 (4.6 × 150 mm, 2.6 μm particle size) column and an Accucore Defender Guard cartridge as pre-column. The running method was as follows: the flow rate of 0.5 mL/min with a gradient from deionized Milli-Q water + 0.1% of formic acid (A), /HPLC grade acetonitrile + 0.1% of formic acid (B) 95:5 to 87:13 over 25 min, then the amount of B was increased to 15% during 1 min and an isocratic elution was maintained during 9 min. The B concentration was then increased to 40% over 5 min and kept constant for 10 min. Before the following new sample injection, the B concentration was lowered again to 5% in 5 min and isocratic elution at A/B 95:5 was further maintained during 10 min. Semi-preparative HPLC was performed using a RP C18 interchrom interchim modulo-cart strategy column (10 × 250 mm) with a Thermo Scientific SpectraSystem P1000XR instrument (Life Technology SAS, Villebon-sur-Yvette, France) and an ultraviolet detector Thermo Scientific SpectraSystem UV1000 (Life Technology SAS, Villebon-sur-Yvette, France) for the monitoring at 310 nm. The following method was optimized for full *p*-nitrophenyl β-d-galactofuranosyl-(1→X)-α-d-mannopyranoside regioisomers separation (X = 2, 3, 4 or 6; these disaccharides are referred as G(1→X)M). The elution proceeded at 7 mL/min. First, a gradient from A/B 80:20 to 75:25 over 30 min was performed, then an isocratic elution was maintained during 100 min, then the amount of B was increased to 80% over 20 min and finally a new isocratic elution was maintained for 20 min. Fractions were assessed using the analytical HPLC method before pooling and freeze-drying. Spectroscopic screenings were performed in a Microplate Spectrophotometer Powerwave XS/XS2 (BioTek France, Colmar, France) and the data were evaluated with the BioTek Gen5 data analysis software (BioTek France, Colmar, France). Plasmid Ysbl-LIC-pET28a containing *ctaraf51* WT gene (Gene ID: 4809304) was a generous gift provided by Prof. G. Davies, University of York, York, UK.

### 3.2. Activity Units

One unit of activity corresponds to the amount of enzyme releasing 1 µmol of *p*-nitrophenol per minute when incubated at 60 °C with Gal*f*-*p*NP 2 mM in a 100 mM phosphate buffer. pH 7. *p-*nitrophenol concentrations were assessed through absorbance measurement at 405 nm (monitoring of phenolate species) due to an appropriate standard curve using pure *p-*nitrophenol set up in same conditions.

### 3.3. Mutagenesis and Screening

#### 3.3.1. Random Mutagenesis

Random mutagenesis was performed by the GeneMorph II Random Mutagenesis kit (Stratagene) using mutagenic PCR. The open reading frame *ctaraf51* encoding the *Ct*Ara*f*51 enzyme was amplified using the following primers: forward T7 promoter TACGACTCACTATAGGGGAA and reverse T7 terminator GCTAGTTATTGCTCAGCGGT.

For a low mutation rate (mutation frequency 0–4.5 mutations/kb), 637 ng of the previously amplified *ctaraf51* gene were mixed with 0.5 µL of each primer (solution at 250 ng/µL); 1 µL of 40 mM dNTP mix (final concentration of 200 µM each), 5 µL of 10 × Mutazyme II reaction buffer and 1 µL of Mutazyme II DNA polymerase (2.5 U/µL) completed to 39 µL with distilled H_2_O. The reaction was thermocycled (MJ Mini Personal Cycler Bio-Rad) as follows: one hot initial denaturation cycle (95 °C, 2 min) then 10 cycles; first the denaturing step (95 °C, 30 s), the annealing step (60 °C, 30 s) and the elongation step were performed for 1 min/kb (72 °C, 3 min); followed by final extension at 72 °C for 10 min. Mutagenesis PCR products (or WT *ctaraf51* PCR-amplified gene) were directly inserted into a plasmid vector pCR^®^2.1-TOPO^®^ (3.9 kb) from Invitrogen (Life Technology SAS, Villebon-sur-Yvette, France) following the TOPO^®^ Cloning protocol provided by the manufacturer. The bank of plasmid was first transformed into home-made *E. coli* XL1 blue competent cells and plated on LB/agar medium. All the resulting colonies were scraped and cultivated over night at 37 °C and 250 rpm in a liquid LB (100 μg/mL ampicillin) medium. Plasmid extraction with the Promega^®^ Wizard Plus SV Miniprep DNA Purification System (Promega France, Charbonnières-les-Bains, France) was performed following the manufacturer’s protocol, and another transformation was achieved using home-made *E. coli* BL21(DE3) competent cells with the purified constructs pCR^®^2.1-TOPO^®^-*ctaraf51-*mutants or the construct pCR^®^2.1-TOPO^®^-*ctaraf51*-WT.

#### 3.3.2. Pre-Screen on Solid State Medium

BL21(DE3) transformed cells (350 µL) were plated on a 20 × 20 cm nitrocellulose membrane (pore size 0.45 µm, Protran, Whatman^TM^, Cytiva Europe GmbH, Velizy-Villacoublay, France) which was on a square Petri dish filled with 150 mL solid LB agar supplemented with 100 µg/mL of ampicillin. The plate was incubated at 37 °C overnight. The nitrocellulose membrane was transferred onto another plate with a minimal medium solid agar (110 mL of sodium phosphate buffer 100 mM pH 7, agar 15 g/L, 100 µg/mL of ampicillin, 5-BI-Gal*f* at 0.5 mM and spread with an isopropyl β-d-1-thiogalactopyranoside (IPTG) solution (5 mL at 50 µM)). After a 3 h incubation at 30 °C, colonies displaying a pale and medium blue color were selected by visual inspection.

#### 3.3.3. Screening Methodology

All the selected variants were picked and transferred to sterile 96 deep-well plates filled with 1 mL of LB medium containing 100 µg/mL ampicillin. After 65 h of growth at 37 °C with horizontal shaking (250 rpm) in an incubator shaker (Thermo Scientific^TM^ MaxQ^TM^ 4000 Shaker, Life Technology SAS, Villebon-sur-Yvette, France) the microplates were replicated to inoculate cultures in 96 deep-well sterile plates (1.5 mL LB supplemented with 100 µg/mL ampicillin per well) grown for 20–24 h at 37 °C under horizontal shaking (250 rpm). When cells were grown to mid-exponential phase (OD _600 nm_ = 0.5), IPTG was added to a final concentration of 0.5 mM and the cultures were incubated for further 18 h at 37 °C. The plates were then centrifuged (3000× *g*, 1 h 30, 4 °C), the supernatant was removed and the pellets were resuspended in 500 µL of 100 mM phosphate buffer supplemented with lysozyme (0.1 mg/mL). A 30 min incubation at 37 °C under agitation was then applied and cultures were frozen at −80 °C for 12 h. After thawing for 1 h at 50 °C, the deep-well plates were centrifuged (3000× *g*, 1 h 30min, 4 °C). The supernatants were kept at 4 °C until use and referred as enzymatic extracts.

In sealed 96-well microtiter plates, 60 µL of each enzymatic extract were incubated with Gal*f-*octyl donor (10 mM) and acceptor Man*p*-*p*NP (60 mM). The reactions were performed in phosphate buffer pH 7, (final volume of 200 µL) and at 60 °C under stirring (200 rpm, Thermo Scientific^TM^ MaxQ^TM^ 4000 Shaker). A total of 50 µL aliquots of each well were retrieved at 1 h and 30 min, 3 h and 17 h, diluted with an equal volume of deionized water and placed at 100 °C for 20 min for enzyme denaturation. The set of samples was stocked at 4 °C until HPLC analysis.

### 3.4. General Procedure for Time Course Monitoring of Enzymatic Assays

A mixture of 10 mM Gal*f*-octyl and 60 mM Man*p*-*p*NP in 100 mM of potassium phosphate buffer pH 7 was preheated at 60 °C and continuously shook at 1400 rpm (Eppendorf Thermomixer^®^ compact, Eppendorf France SAS, Montesson, France). Enzyme solutions were added to the preheated and sonicated substrate solutions at t = 0 of kinetics. Aliquots (50 µL) of the reaction mixture were withdrawn at different times, and quenched with an equal volume of acetonitrile. The set of samples was stocked at −20 °C until HPLC analysis.

### 3.5. Preparative Scale Synthesis of G(1→X)M Disaccharides

A solution of 150 mg (10 mM) of donor Gal*f*-octyl and 900 mg (60 mM) of acceptor Man*p*-*p*NP in 100 mM phosphate buffer pH 7 was heated at 60 °C and sonicated until complete dissolution. A phosphate buffer solution of *Ct*Ara*f* 51 was added to reach 392 U/µmol of donor and the reaction was heated at 60 °C while stirring. The mixture was quenched in liquid nitrogen, lyophilized and submitted to a 2-step purification: separation on silica gel chromatography (CombiFlash) to discard *p*-nitrophenol and most of the Man*p*-*p*NP excess with a gradient of ethyl acetate/(*i*PrOH:H_2_O; 50:50) 97:3 to 50:50. After solvent evaporation and freeze-drying, the resulting enriched regioisomer mixture was solubilized in deionized water at a concentration of 10 mg/mL. Runs were performed on 1 mL aliquots that were submitted to semi-preparative reverse-phase C18 HPLC column. The 4 G(1→X)M regioisomers were isolated and characterized by ^1^H and ^13^C NMR and HRMS (details in Supplementary Materials).

### 3.6. Molecular Modelling

#### 3.6.1. Computational Methods and Details

The simulations were performed on a PC workstation with an Intel^®^ Xeon Silver 4214 CPU 2.20 GHz (Santa Clara, CA, USA), or a laptop PC Dell Latitude 7520 with an Intel^®^ Core^TM^ i7-6820HQ CPU 2.70 GHz and a Mac Os station Core i5. Molecular docking and dynamics were carried out via the Yasara 19.1.27 software interface [17]. The crystal structure of the arabinofuranosidase 51 from *Rumini Clostridium thermocellum (Ct*Ara*f*51*)* subunit in interaction with its ligand α-l-arabinofuranosyl-(1→3)-α-d-xylopyranose (PDB ID: 2C8n) was initially used [10].

#### 3.6.2. Preparative Molecular Dynamics on Glycosyl-Enzyme

On the basis of our previous computational investigations on the *p*-nitrophenyl α-l-arabinofuranoside and Gal*f*-*p*NP interactions with *Ct*Ara*f*51 subunit [9], galactofuranosyl-enzyme covalent complex was built on the nucleophilic glutamate E292 with the Yasara Structure module, and optimized by Restricted Hartree–Fock methods [18]. Geometrical optimizations and energy minimizations were performed by semi-empirical method AM1, coupled with the YAMBER3 force field [19], an AMBER version for Yasara. In periodic conditions, a first global optimization was executed in aqueous environment (ε = 80) adjusted at pH 7, inside a cell including 5 Å around all atoms of the covalent complex. Long range electrostatic interactions were calculated using the Particule Mesh Ewald (PME) algorithm [20]. A 10.5 Å cut off was defined for Van der Waals and Coulomb interactions to compute potential energy [21]. Yasara methods relative to hydrogen orientation optimizations and hydrogen bonding network calculations kept the same main approach from the WHAT IF software [22] with additional features, particularly for the protonation states in proteins and ligand [23]. Silico mutants of residues S214, E225, D327 were built and optimized with the same conditions.

In the first stage, molecular dynamics (MD) were carried out, for comparison, on the Gal*f-*WT enzyme complex and the different mutants to investigate conformational evolutions of the covalent complexes. MD simulations were developed at 298 K, using the same force field, in a similar cell around the glycosyl-enzyme with Yasara package dynamics [24]. The solvation cell was filled with water molecules, according to a solvent density of 0.997 g/mL. All the atoms were surrounded by 5 Å water molecules and the system charge was neutralized, using NaCl with 0.9% concentration to maintain the pH at 7 over the entire simulation period. The MD simulations were run for 20 to 25 ns in periodic boundaries at constant volume with multiple time-step 1.25 fs to 5 fs [25]. Preliminary energy minimization by steepest descent was performed to remove severe bumps, followed by simulated annealing minimizations at 298 K and velocities were scaled down every ten steps for a total time of 5 ps in 500 steps and to a final temperature of 0 K. Equilibration time and simulation trajectories were visualized and studied by Yasara analysis algorithms. Last, the DM Gal*f*-*Ct*Ara*f*51 complexes were kept to achieve the energetically favored positions of the Gal*f* ring in the active site. These Gal*f-*WT enzyme or mutants complexes were compared considering distances from anomeric oxygen of glycosyl-E292 residue to glutamate E173. Hydrophobic interactions, hydrogen bonds, specific interactions from catalytic or key residues were also compared. The particular locations and motions of S214 and D327 residues and their mutants were examined with calculations of root mean square fluctuations (RMSF) and B-factor analysis maps per-residue [26].

#### 3.6.3. Local Dockings of Disaccharides and Elaboration of Manp-pNP Complexes

Preliminary global dockings [27], then local dockings, were developed using Autodock Vina [28] by Yasara, according the same force field and same previous conditions of energy minimizations.

In the second stage, taking into account the foregoing computational studies on an α-l-Fucosidase by Tellier’s group [29], extensive local docking studies were implemented in order to investigate the (1→3), (1→4), (1→6) regioselectivity of the Man*p*-*p*NP glycosidic approaches.

The models of disaccharide ligands were built from Glycam-web carbohydrate builder [30] and improved optimizations were performed using Gaussian v9 software [31] with a standard B3LYP functional combined with the 6-311+G** basis set [32,33].

So, each β-d-Gal*f*-(1→X)-α-d-Man*p-p*NP product was inserted into a cell (10 to 12 Å) centered on nucleophilic and acid base glutamate atoms (E292 and E173, γ-COO) of a wild-type enzyme or mutant. The cell size was adjusted to include mutated residues. After local optimization, all β-d-Gal*f*-(1→X)-α-d-Man*p-p*NP disaccharides were docked on the active site of *Ct*Ara*f*51 and mutants in wall boundaries.

Then, each docked disaccharide complex was superimposed to the corresponding last MD Gal*f* wild-type enzyme or mutant complex. From the docking of the disaccharide, only the Man*p-p*NP part of the disaccharide was conserved and fused to the Gal*f* glycosyl-enzyme to build an initial glycosyl-enzyme complex with the acceptor in the considered (1→X) orientation. Subsequently, each complex Gal*f* wild type or mutant with Man*p*-*p*NP was optimized and validated by local redockings [34] on the basis of suitable relative binding energy and dissociation constant evaluations and an acceptable distance from Gal*f* anomeric carbon to the considered hydroxyl group of the Man*p*-*p*NP.

#### 3.6.4. Molecular Dynamics of Glycosyl-Enzyme Complexes with Manp-pNP

In the third stage, MD simulations were performed to probe the stability and the evolution of each docking complex Gal*f* glycosyl-enzyme with a specific (1→X) orientation of the acceptor Man*p*-*p*NP*,* during 25 to 30 ns. All complexes remained free (no rigid part) in particular at the galactofuranosyl ring, to consider distortions during the acceptor approach. MD were performed in the same previous conditions used for MD with the Gal*f*-*Ct*Ara*f*51 covalent complexes.

Simulation trajectories were analyzed following standard parameters as well as specific distances or relevant angles with the different (1→X) envisioned glycosidic linkages. In silico binding energy evolutions between complexes and Man*p*-*p*NP were analyzed for each MD simulation.

So, MD conformation with the strongest binding energy and the equilibrium last conformation were compared with regards to the potential minimal energy conformation. For internal validation of MD simulations on a wild-type enzyme and its mutants, these conformations were superimposed with RMSD calculations.

The last MD and strongest binding energy conformations were studied to determine potential congruence between transglycosylation and conformational changes. So, a specific angle or distance such as between considered the hydroxyl group to the Gal*f* anomeric carbon, and to the E173 glutamate were compared on these conformations. The location of the Man*p*-*p*NP ligand and the position of Gal*f* ring were also examined considering their interactions (hydrogen bonds, hydrophobic, π-π, π-stacking interactions, and cation-π interactions) and also particular carbohydrate recognition interactions (π-CH interactions… [35]) with the enzyme or the mutant. The motions and interactions of mutated residues were also considered for their potential effects on conformational change in the acceptor binding site.

Interpreting the flexibility and dynamics, B-factor represents both vibrations and static disorder. So, at best, several temperature measurements for separating these two effects should be made. Ideally, high-resolution X-ray structures are necessary for deriving reliable B-factors. So here, a careful use of B-factor correlated with RMSF was developed [36] (Supplementary Materials Figures S5 and S6).

## 4. Conclusions

The synthesis of disaccharides using enzymatic means appears to be a good alternative compared with traditional glycochemistry. Glycosidases are interesting options as they can use easily accessible substrates. Two main difficulties need to be considered: their transglycosylation over hydrolysis balance, and their stereochemistry. The transglycosylation over hydrolysis balance can be improved either by the reaction settings or by modifications of the catalyst. Two main approaches were applied for the latter: mutation of the nucleophilic residue to obtain glycosynthases, or mutations apart from the catalytic residues to modify the hydrolysis over transglycosylation balance without altering the overall mechanism. Glycosynthases are very efficient tools for that purpose [37] but with their altered mechanism, they require activated donor substrates (mainly glycosyl fluorides), meaning additional steps of tedious glycochemistry in the overall process [38]. On the other hand, improving the transglycosylation over the hydrolysis ratio without modifying the catalytic residues remains a difficult task. Such improvements were performed with a few enzymes [12,39,40,41], but except for enzymes from the GH1 family, where water channels were identified and modified to alter specifically the hydrolysis [11,42], these modifications are difficult to rationalize and to extend to other enzymes. Recently, a strategy focused on the modifications of conserved residues within a GH family appeared very promising to improve the transglycosylation efficiency [43], yet the control of the regioselectivity remains an important parameter to focus on. To generate additional data on this field, a random mutagenesis approach combined with a tailored modelling methodology were performed on the arabinofuranosidase *Ct*Ara*f*51 in order to highlight critical residues involved in this balance and in the regioselectivity of the enzyme. By comparing pre-selected mutants, based on the assumption that improved transglycosylation generally results in an overall lower activity, in the presence or absence of acceptor substrate, we were able to isolate two very promising mutants, with a 2.3 to 3.8-fold increase in transglycosylation yield. The screening methodology did not allow direct discrimination of mutants with modified regioselectivity, but it appeared that these two mutants had very different regioisomer kinetic profiles compared with the wild type. Mutations of the MYC98 mutant were close to the active site and *in silico,* our tailored methodology enabled the rationalization of direct and local conformational modifications of the active site on the regioselectivity. MYC80 mutation was far from the active site, nonetheless molecular dynamics and B-factor analysis allowed observations of remote effects on the interactions with the acceptor. In summary, S214T and D327N were identified as promising mutations to improve the *Ct*Ara*f*51 transglycosylation capability with modified regioselectivity. Moreover, the prospect S214T and D327N coupled mutations must be considered in order to improve further transglycosylation and regioselectivity of the enzyme. With a good agreement between the experimental transglycosylation kinetics and in silico investigations, this work highlighted new biocatalysts for eco-friendly syntheses of attractive disaccharide β-d-galactofuranosyl-(1→3)-d-mannopyranose and its (1→4) counterpart. This in silico methodology enables then a rational approach to study modulations of the glycosylation regioselectivity and are now of great use for further studies of regioselectivity modulation with other model enzymes.

## Figures and Tables

**Table 1:** Identified mutations of mutants with IF > 2.

Mutant Name	Mutations
MYC44	V58M, F255L, E452K
MYC69 = MYC70 = MYC98	E80G, S214T, E225D, L451M, K503N
MYC80	D327N

**Table 2:** MD simulation parameters to study (1→X) regioselectivity of Man*p*-*p*NP approaches. Distances a_X_ are depicted in Scheme 2. When shorter distances a_X_ were observed with other hydroxyl (OH) than the initial one, they were added between brackets.

	Distances a_X_ (Å)		
Initial (1→X) Orientation	X = 3	X = 6	X = 4
*Ct*Ara*f* 51-wild type	5.60	3.33	6.74 [6.10 (X = 3)]
*Ct*Ara*f* 51-S214T mutant	4.16	6.78 [3.72 (X = 3); 3.48 (X = 4)]	3.88 [3.37 (X = 3)]
*Ct*Ara*f* 51-S214T-E225D (MYC98)	4.60	7.57	3.55 [3.64 (X = 3)]
*Ct*Ara*f* 51-E225D (MYC98-T214S)	5.37	3.51	8.12 [5.37 (X = 3)]
*Ct*Ara*f* 51-D327N (MYC80) mutant	3.95	4.10 [3.30 (X = 4)]	3.36

## Data Availability

The data presented in this study are available in the article.

## Supplementary Materials

The following are available online. Additional figures and tables: Figure S1: Transglycosylation between Gal*f*-octyl (10 mM) and Man*p*-*p*NP (20–100 mM) with different donor/acceptor ratios. Figure S2. Transglycosylation improvement factors of the 120 screened mutants. Table S1: parameters a_X_, b_X_ and α_X_ of MD complexes to study Man*p*-*p*NP orientations approaching Gal*f*-enzyme intermediates. Figure S3: Gal*f-*enzyme intermediates interacting with Man*p*-*p*NP after MD starting from the different (1→X)-oriented Man*p*-*p*NP (X = 3, 6, 4; enzyme = *Ct*Ara*f*51 WT, *Ct*Ara*f*51 S214T, *Ct*Ara*f*51 S214T E225D, *Ct*Ara*f*51 E225D, *Ct*Ara*f*51 D327N). Figure S4: Secondary structure of *Ct*Ara*f*51 wild type and selected mutants. Figure S5: B-Factor maps per residue on MD complexes for *Ct*Ara*f*51 WT-Gal*f* and *Ct*Ara*f*51 S214T-Gal*f* intermediates interacting with Man*p*-*p*NP according to its initial (1→X)-orientation (X = 3, 6, 4). Figure S6: B-Factor maps per residue on MD complexes for *Ct*Ara*f*51 WT-Gal*f* and MYC80 [D327N]-Gal*f* intermediates interacting with Man*p*-*p*NP according to its initial (1→X)-orientation (X = 3, 6, 4). Supplementary Experimental part: SE1: Synthesis of the donor substrate 5-bromo-indolyl β-d-galactofuranoside (5-BI-Gal*f*). SE2: G(1→X)M disaccharides characterizations.

## Sample Availability

Samples of the compounds are available from the authors.
