# Supplementary material for: Modulation of the Activity and Regioselectivity of a Glycosidase: Development of a Convenient Tool for the Synthesis of Specific Disaccharides
Source: Molecules. 2021 Sep 7;26(18):5445. doi: 10.3390/molecules26185445 (PMC8468180; doi:10.3390/molecules26185445)
Supplement: Supplementary file 1 [file molecules-26-05445-s001.zip › molecules-1299281-supplementary.pdf]

Modulation of the activity and regioselectivity of a glycosidase: development of a convenient tool for the synthesis of specific disaccharides.

Supplementary Informations

Yari Cabezas-Pérusse,[a] Franck Daligault,[b] Vincent Ferrières,[a] Olivier Tasseau,[a] Sylvain Tranchimand.[a*]

[a] Université de Rennes, Ecole Nationale Supérieure de Chimie de Rennes, CNRS, ISCR (Institut des Sciences Chimiques de Rennes) – UMR 6226, F-35000 Rennes, France.

[b] Université de Nantes, CNRS, UFIP (Unité de Fonctionnalité et Ingénierie des Protéines) - UMR 6286, F-44000 Nantes, France.

[*] Author to whom correspondence should be addressed.

Table of content

Additional figures:

- **Figure S1:** Transglycosylation between Gal*f*-octyl (10 mM) and Man*p*-*p*NP (20-100 mM) with different donor/acceptor ratios. 3

- **Figure S2:** Transglycosylation improvement factors of the 120 screened mutants. 3

- **Table S1:** parameters a_X, b_X and α_X of MD complexes to study Man*p*-*p*NP orientations approaching Gal*f*-enzyme intermediates........ 4

- **Figures S3**: Gal*f*-enzyme intermediates interacting with Man*p*-*p*NP after MD starting from the different (1→X)-oriented Man*p*-*p*NP (X = 3, 6, 4; enzyme = *Ct*Ara*f*51 WT, *Ct*Ara*f*51 S214T, *Ct*Ara*f*51 S214T E225D, *Ct*Ara*f*51 E225D, *Ct*Ara*f*51 D327N)........ 5

- **Figure S4**: Secondary structure of *Ct*Ara*f*51 wild type and selected mutants. 13

- **Figures S5:** B-Factor maps per residue on MD complexes for *Ct*Ara*f*51WT - Gal*f* and *Ct*Ara*f*51 S214T - Gal*f* intermediates interacting with Man*p*-*p*NP according to its initial (1→X)-orientation (X = 3, 6, 4; enzyme = *Ct*Ara*f*51 WT, *Ct*Ara*f*51 S214T) 14

- **Figures S6:** B-Factor maps per residue on MD complexes for *Ct*Ara*f*51 WT and MYC80 [D327N] - Gal*f* interacting with Man*p*-*p*NP according to its initial (1→X)-orientation (X = 3, 6, 4; enzyme = *Ct*Ara*f*51 WT, MYC80 [D327N]). 20

Supplementary Experimental parts:

- **SE1: Synthesis of the pre-screen donor substrate 5-bromo-indolyl β-D-galactofuranoside (5-BI-Gal*f*)** (adapted from Berlin *et al.*[16]) 26

- **SE2: G(1→X)M disaccharide characterizations.** 27

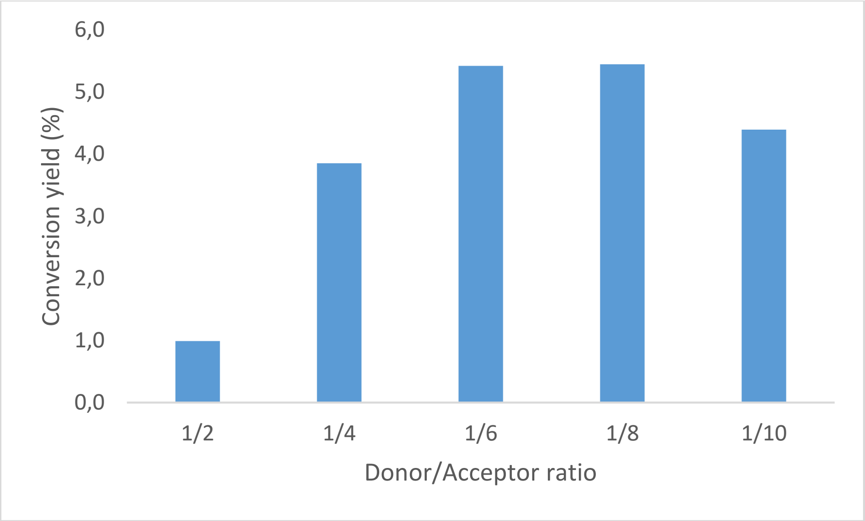

Figure S1: Transglycosylation between Gal*f*-octyl (10 mM) and Man*p*-*p*NP (20-100 mM) with different donor/acceptor ratios catalyzed by *Ct*Ara*f*51 WT at 60°C. The conversion yield is based on the sum of the four G(1→X)M disaccharides after 2 hours of reaction.

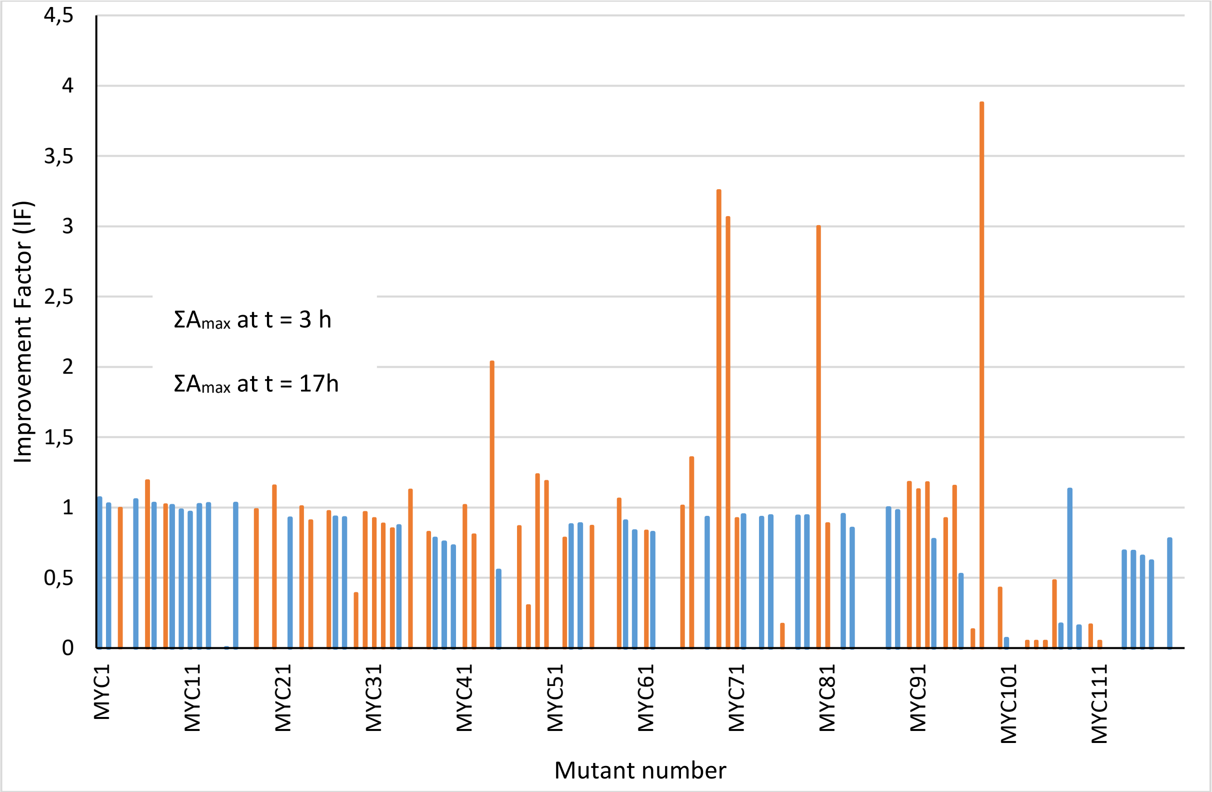

Figure S2: Transglycosylation improvement factors of the 120 screened mutants. The IFs were calculated according to the Equation 1.

Enzyme	Initial *p*NP-Man*p* orientation toward Gal*f*-enzyme	MD complexes	Distances* (a,b) $_3$OH		Distances (a,b) $_4$OH		Distances (a,b) $_6$OH		Angles α_3 ($_3$OH), α_4 ($_4$OH), α_6 ($_6$OH)		
			a_3 (Å)	b_3 (Å)	a_4 (Å)	b_4 (Å)	a_6 (Å)	b_6 (Å)	α_3 (°)	α_4 (°)	α_6 (°)
*Ct*Ara*f*51 WT	$_3$OH	Highest binding energy	5.6	6.3	8.1	7.2	9.1	7.4	148	--	--**
		Final complex	7.4	6.9	9.1	7.9	8.0	6.7	157	--	--
	$_4$OH	Highest binding energy	6.1	5.0	6.7	7.2	9.2	11.2	136	124	--
		Final complex	4.9	8.3	6.2	7.4	10.5	11.7	125	103	--
	$_6$OH	Highest binding energy	7.7	7.5	5.3	7.1	3.3	1.6	--	111	139
		Final complex	7.3	7.5	5.2	7.2	3.5	1.7	--	115	129
*Ct*Ara*f*51 S214T	$_3$OH	Highest binding energy	4.2	6.5	7.0	8.1	9.1	9.0	143	--	--
		Final complex	5.7	7.3	8.1	8.3	8.9	9.2	157	--	--
	$_4$OH	Highest binding energy	3.4	5.6	3.9	5.2	7.0	8.8	116	71	61
		Final complex	3.5	4.0	4.0	5.1	7.1	9.2	136	93	80
	$_6$OH	Highest binding energy	3.7	5.6	3.5	5.4	6.8	9.6	154	105	92
		Final complex	3.2	6.7	3.5	5.3	6.5	9.5	142	92	82
*Ct*Ara*f*51 E225D	$_3$OH	Highest binding energy	5.4	4.7	8.1	8.7	9.8	10.3	150	--	--
		Final complex	5.6	4.6	8.3	8.4	9.7	7.8	155	--	--
	$_4$OH	Highest binding energy	5.4	5.5	8.1	8.5	9.2	10.2	154	143	116
		Final complex	5.5	5.1	8.2	7.9	9.2	7.1	145	135	111
	$_6$OH	Highest binding energy	8.3	8.5	6.7	9.2	3.5	4.1	118	99	91
		Final complex	8.9	9.9	6.8	9.4	4.9	4.3	105	90	101
*Ct*Ara*f*51 S214T E225D	$_3$OH	Highest binding energy	4.6	4.9	7.2	7.2	8.6	7.9	155	--	--
		Final complex	4.7	5.0	7.5	7.5	9.5	9.9	153	--	--
	$_4$OH	Highest binding energy	3.6	4.5	3.5	4.3	7.2	8.7	139	93	84
		Final complex	4.9	5.8	4.2	4.4	7.9	9.4	130	96	82
	$_6$OH	Highest binding energy	7.8	8.3	9.1	8.3	7.6	5.1	152	146	125
		Final complex	8.1	9.3	9.4	9.3	7.9	4.9	140	133	121
*Ct*Ara*f*51 D327N	$_3$OH	Highest binding energy	3.9	4.9	7.0	7.9	9.7	9.0	134	109	--
		Final complex	6.8	6.2	4.1	5.3	5.9	8.1	101	108	--
	$_4$OH	Highest binding energy	6.1	7.7	3.3	6.9	4.1	3.0	94	91	149
		Final complex	6.0	7.8	3.2	6.9	4.1	1.7	104	96	136
	$_6$OH	Highest binding energy	3.8	5.4	3.3	5.1	6.6	8.8	140	94	--
		Final complex	4.0	5.6	3.5	5.3	7.2	9.1	141	93	--

Table S1: Parameters a_X, b_X and α_X of MD complexes to study Man*p*-*p*NP orientations approaching Gal*f*-enzyme intermediates.

* See scheme 3 for distances and angle representations; **(--): atoms were too far, angle α_x has no meaning.

The figures S3 were divided in five parts A_i to E_i corresponding to each enzyme or mutant, (A_i): *Ct*Ara*f*51 WT, (B_i): *Ct*Ara*f*51 S214T, (C_i): *Ct*Ara*f*51 S214T E225D, (D_i): *Ct*Ara*f*51 E225D, (E_i): *Ct*Ara*f*51 D327N. For each of them, the three (i) starting (1→X)-orientations from Man*p*-*p*NP approaches were considered (X = 3, 4, 6).

A1 *Ct*Ara*f*51 Wild type / (1→3)

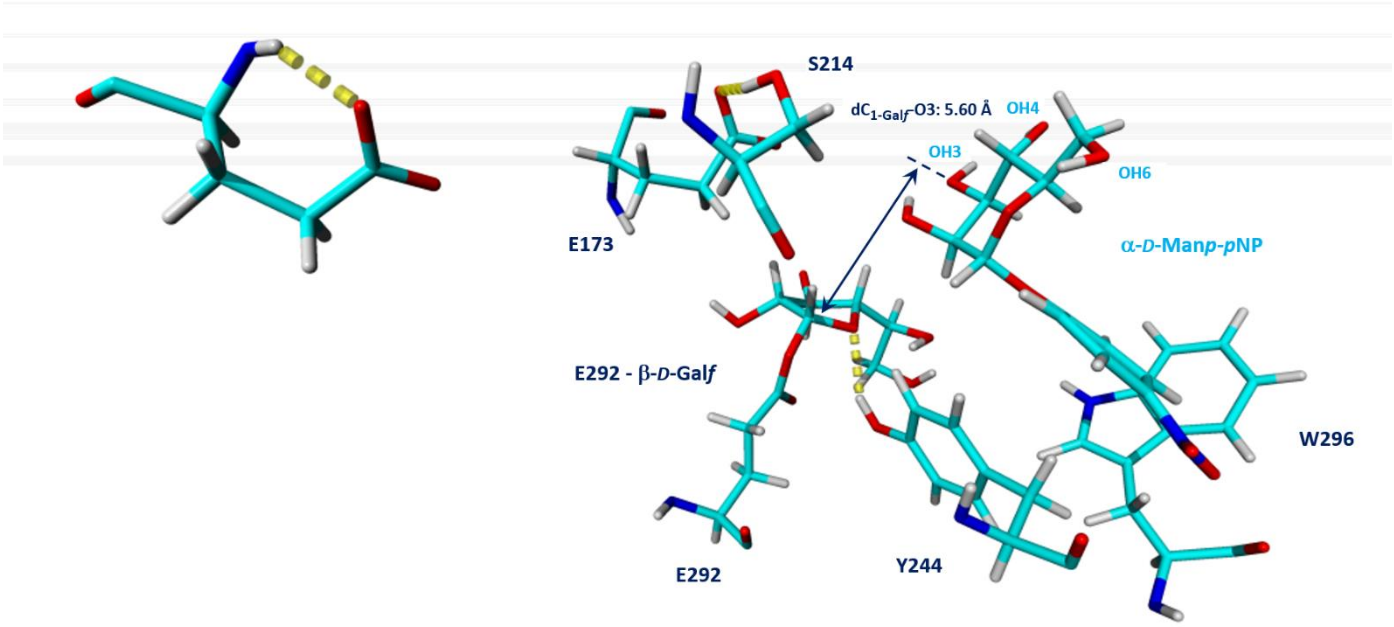

A2 *Ct*Ara*f*51 Wild type / (1→4)

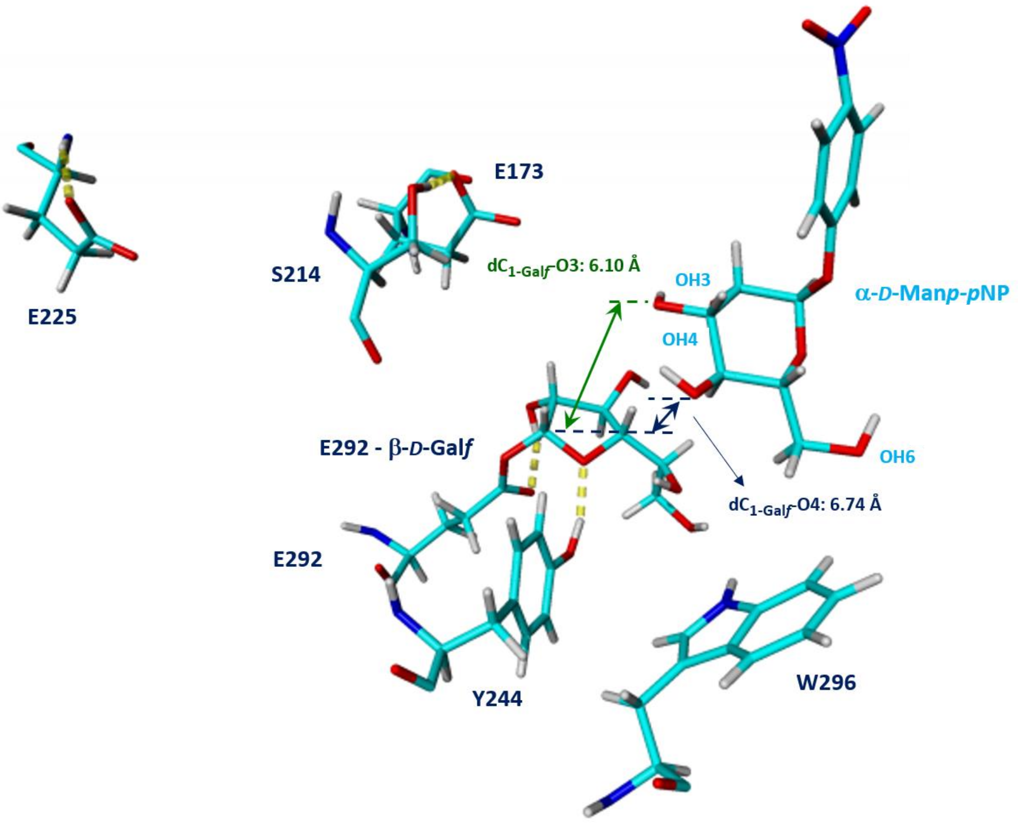

../...

A3 *Ct*Ara*f*51 Wild type / (1→6)

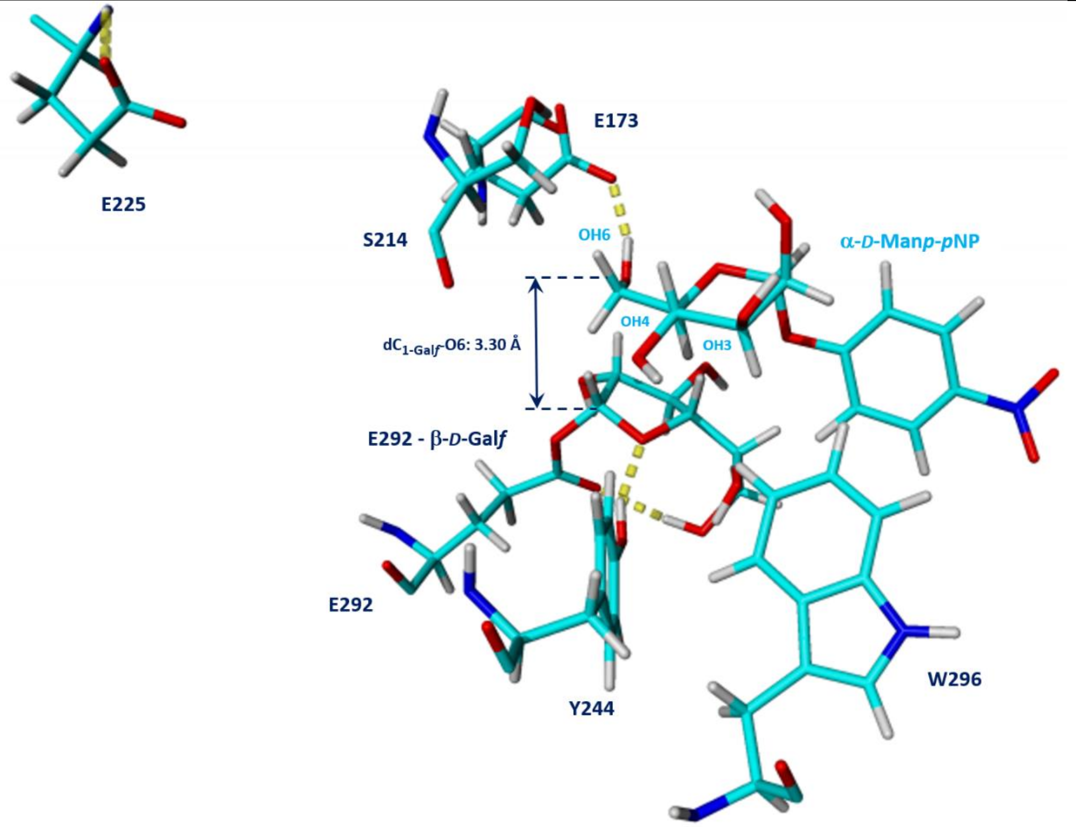

Figure S3(A1, A2, A3): *Ct*Ara*f*51 WT-Gal*f* intermediate interacting with Man*p*-*p*NP after MD starting from the different (1→X)-oriented Man*p*-*p*NP (X = 3, 4, 6).

B1 *Ct*Ara*f*51 S214T / (1→3)

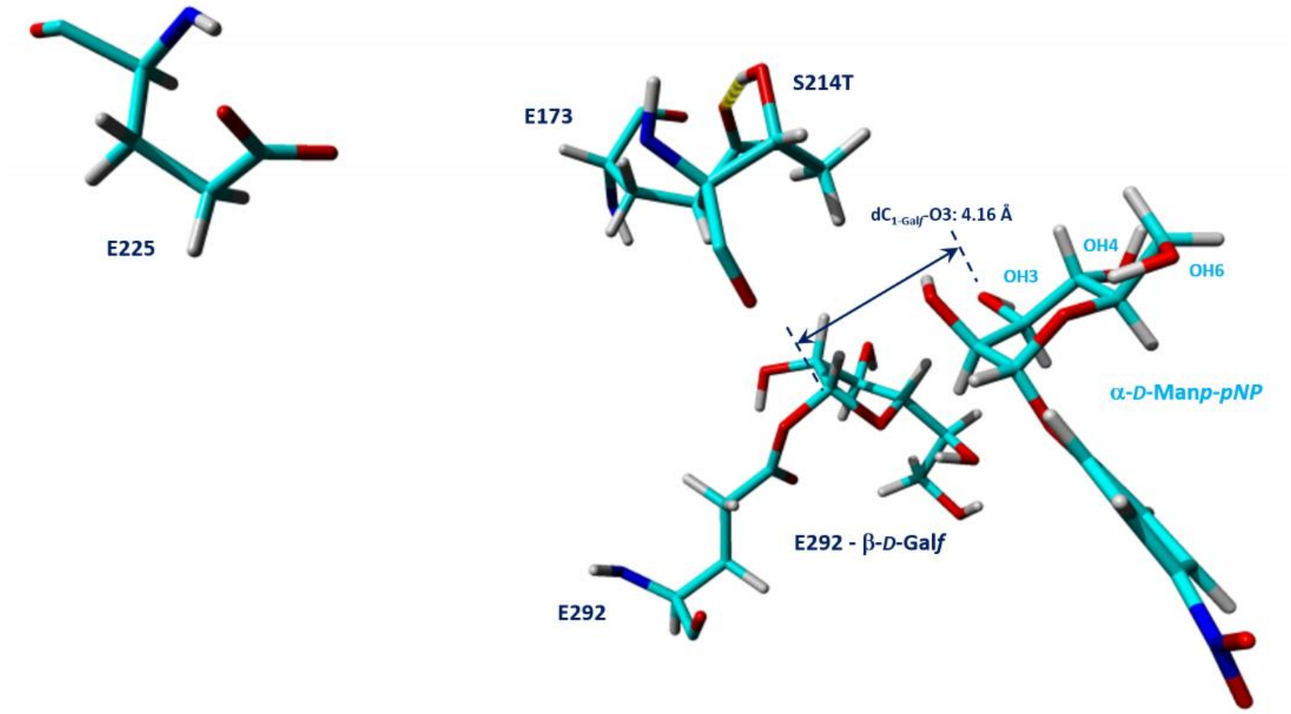

../…

B2 *Ct*Ara*f*51 S214T / (1→4)

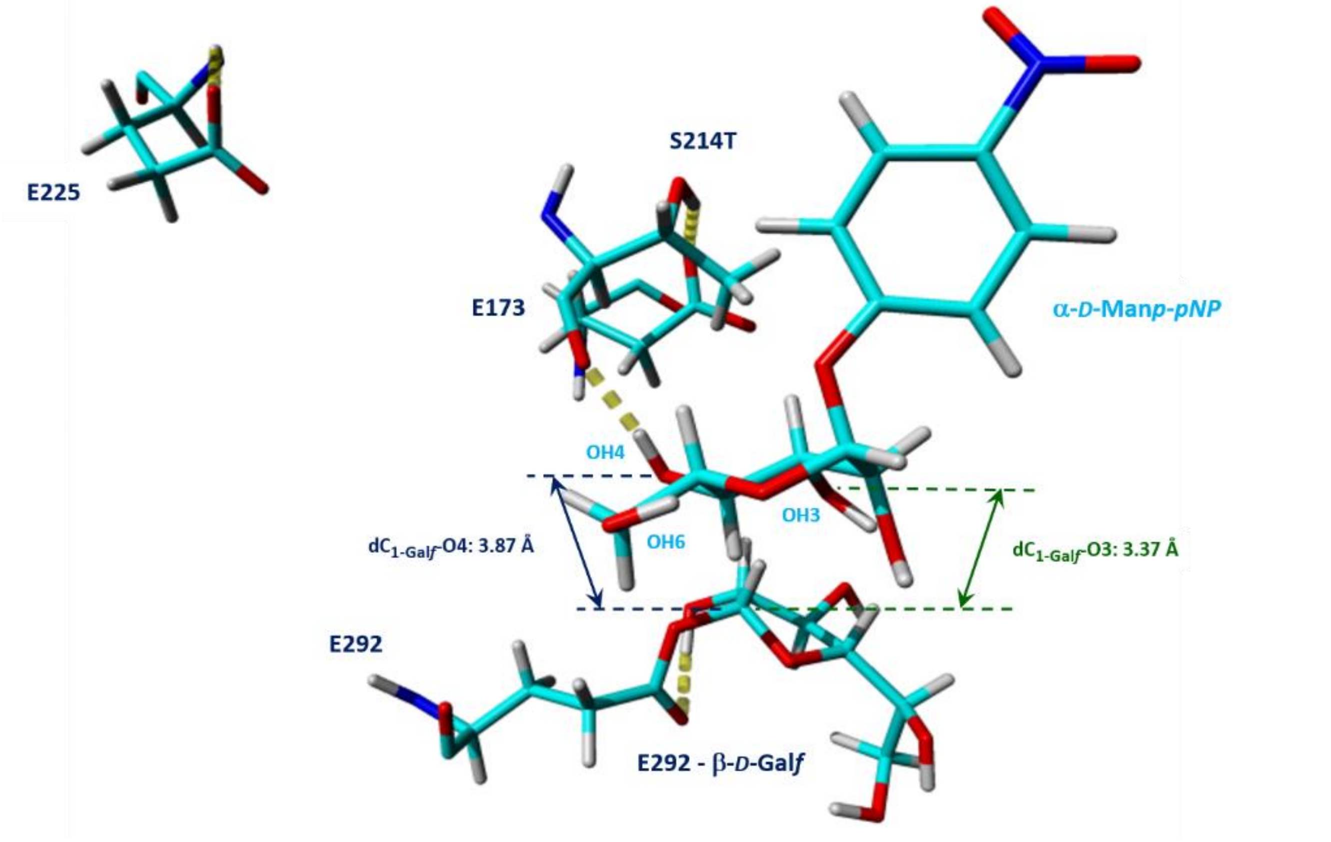

B3 *Ct*Ara*f*51 S214T / (1→6)

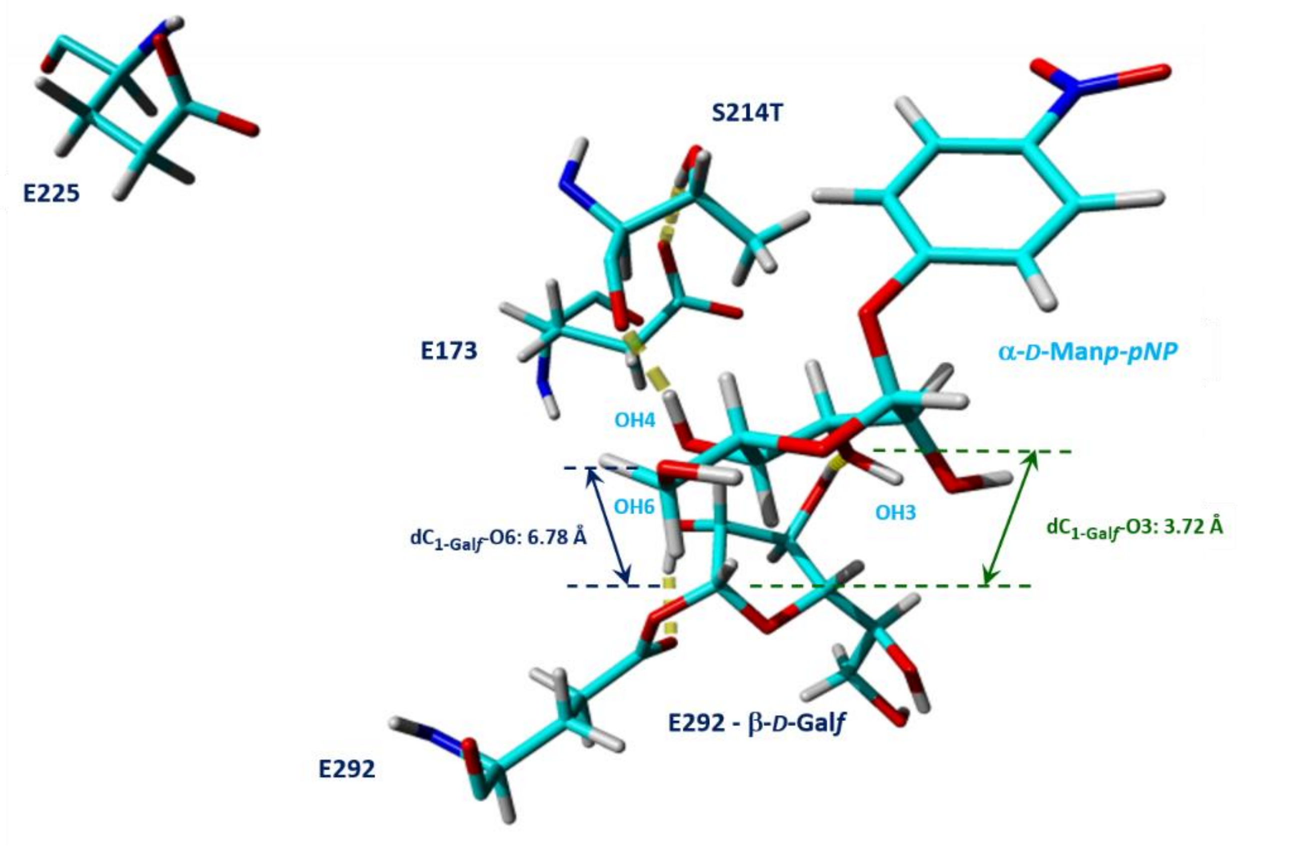

Figure S3(B1, B2, B3): *Ct*Ara*f*51 S214T-Gal*f* intermediate interacting with Man*p*-*p*NP after MD starting from the different (1→X)-oriented Man*p*-*p*NP (X = 3, 4, 6).

C1 *Ct*Ara*f*51 S214T E225D / (1→3)

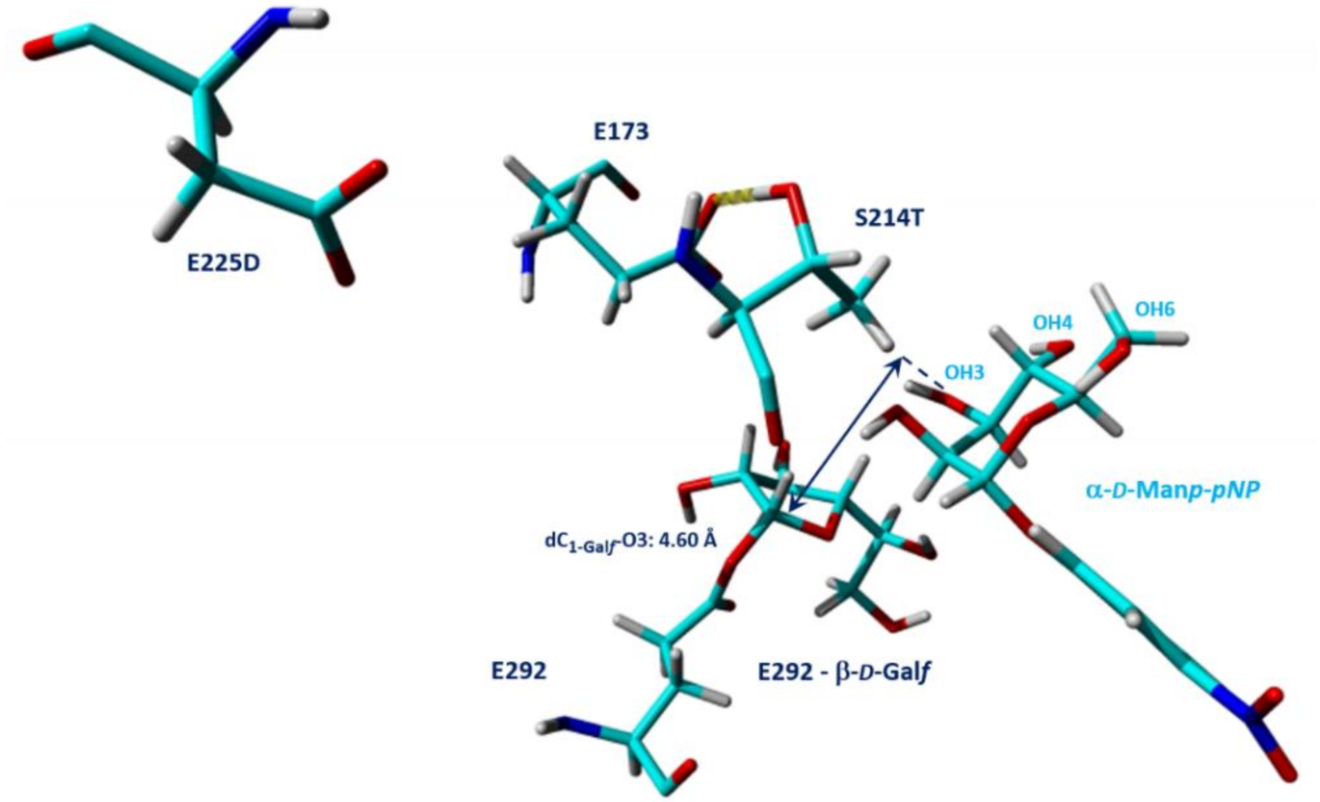

C2 *Ct*Ara*f*51 S214T E225D / (1→4)

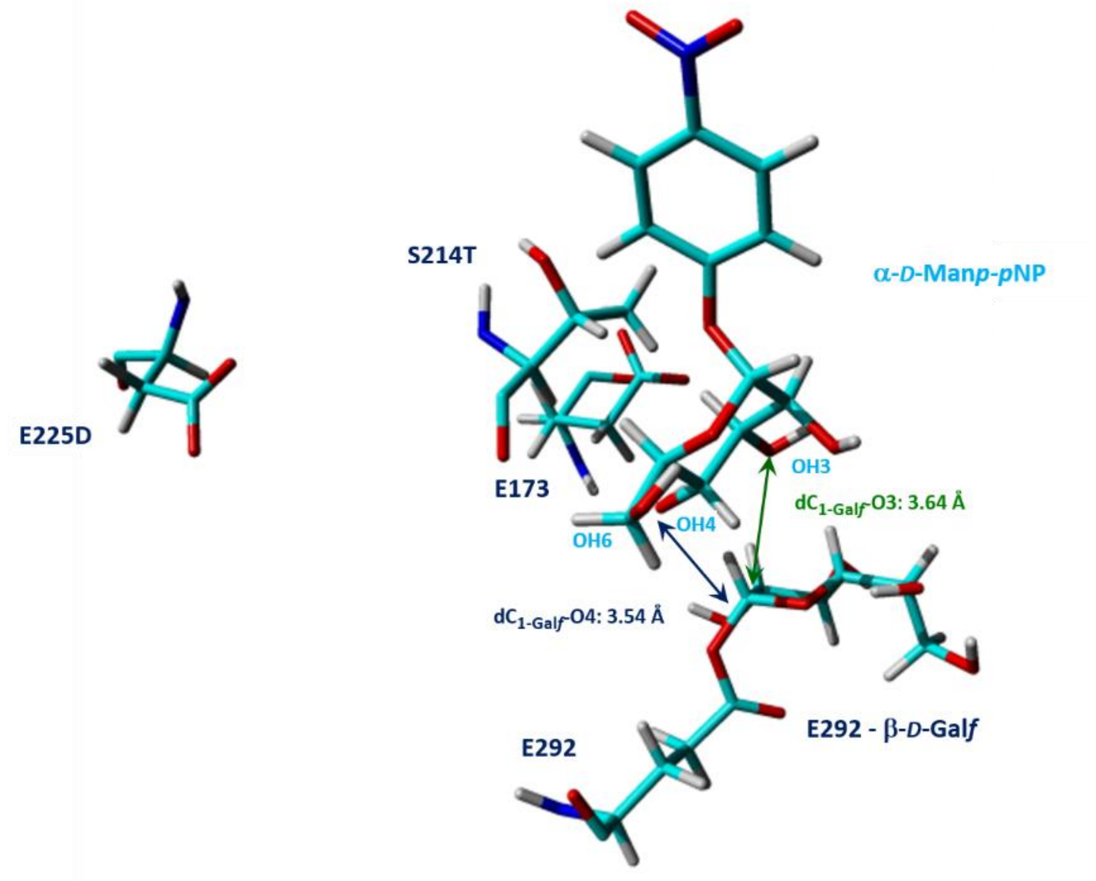

../…

C3 *Ct*Ara*f*51 S214T E225D / (1→6)

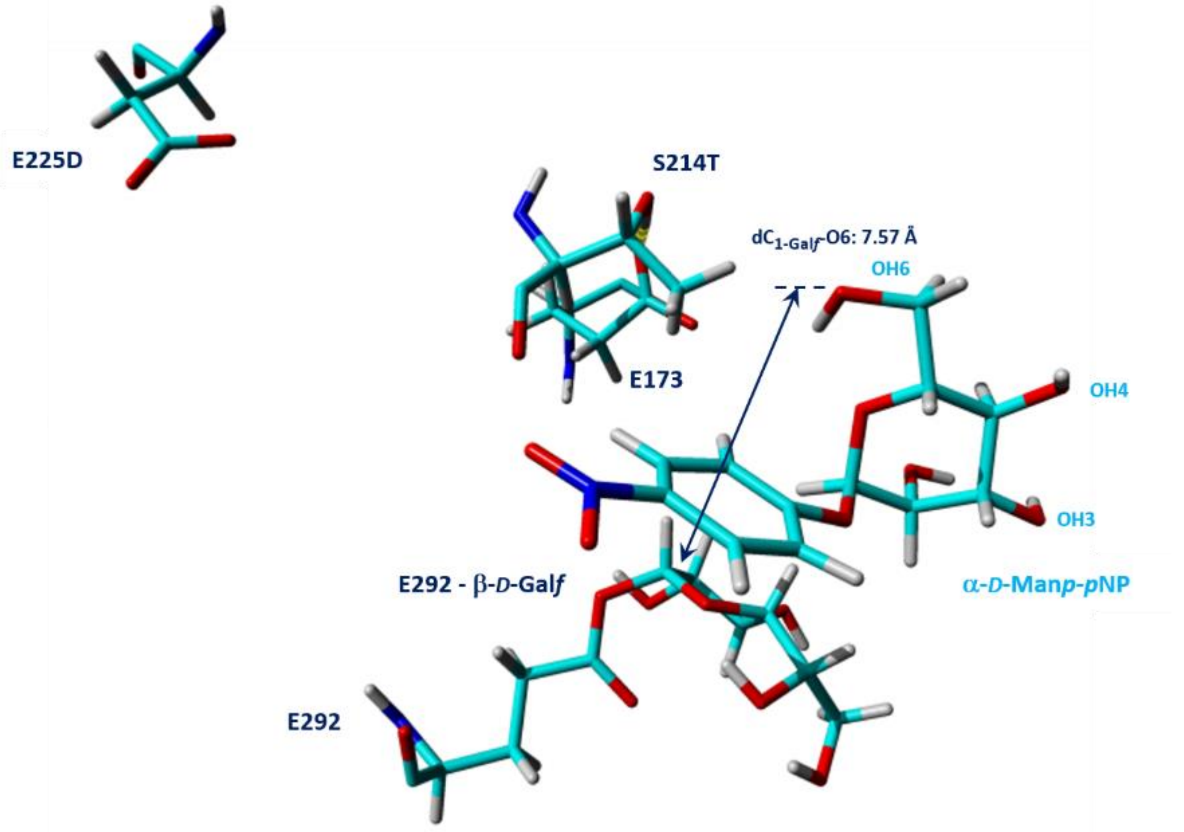

Figure S3(C1, C2, C3): *Ct*Ara*f*51 S214T E225D-Gal*f* intermediate interacting with Man*p*-*p*NP after MD starting from the different *(1→X)*-oriented Man*p*-*p*NP (X = 3, 6, 4).

D1 *Ct*Ara*f*51 E225D / (1→3)

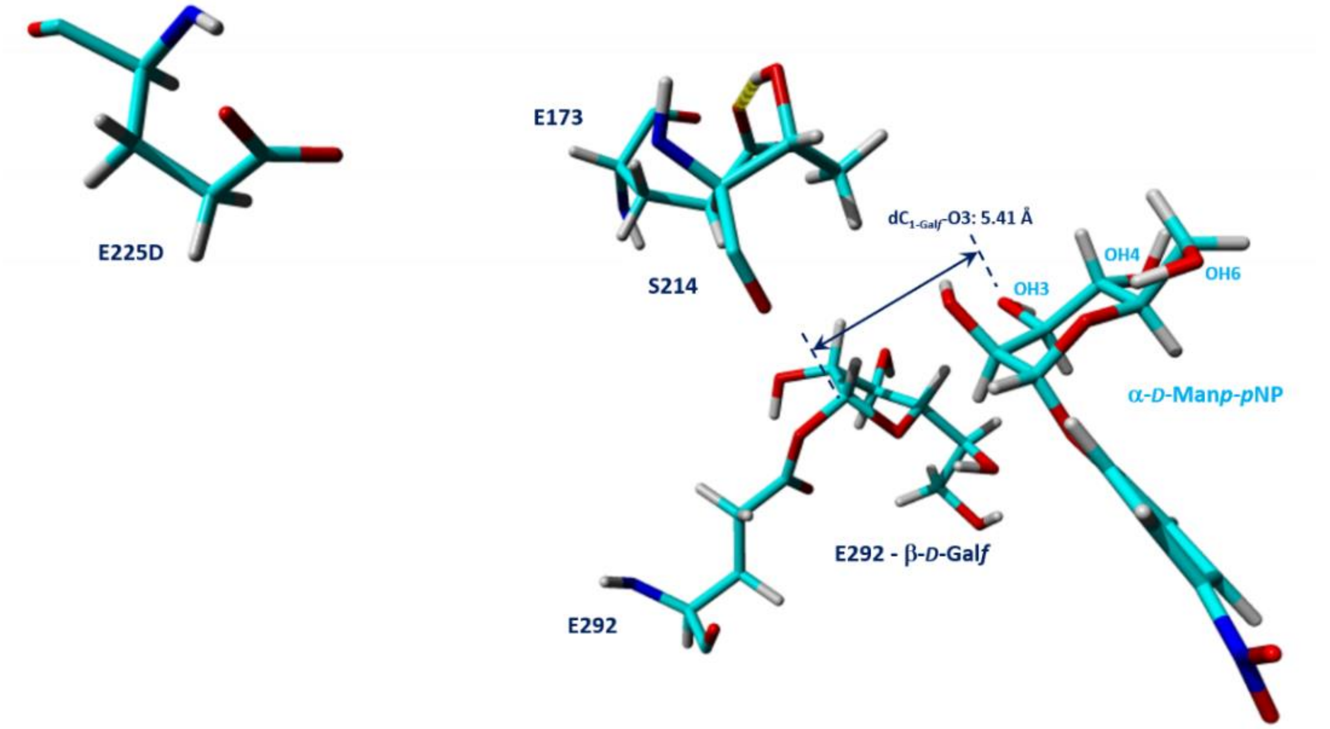

../...

D2 *Ct*Ara*f*51 E225D / (1→4)

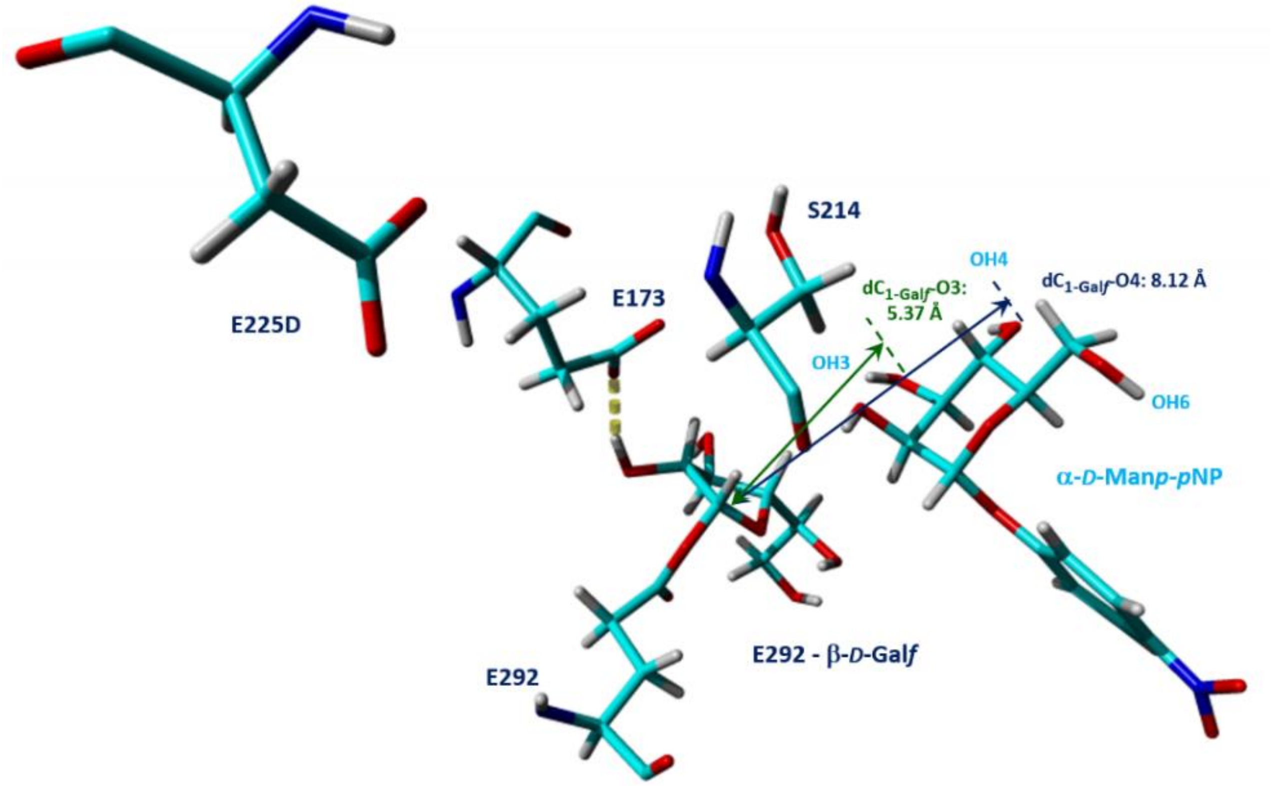

D3 *Ct*Ara*f*51 E225D / (1→6)

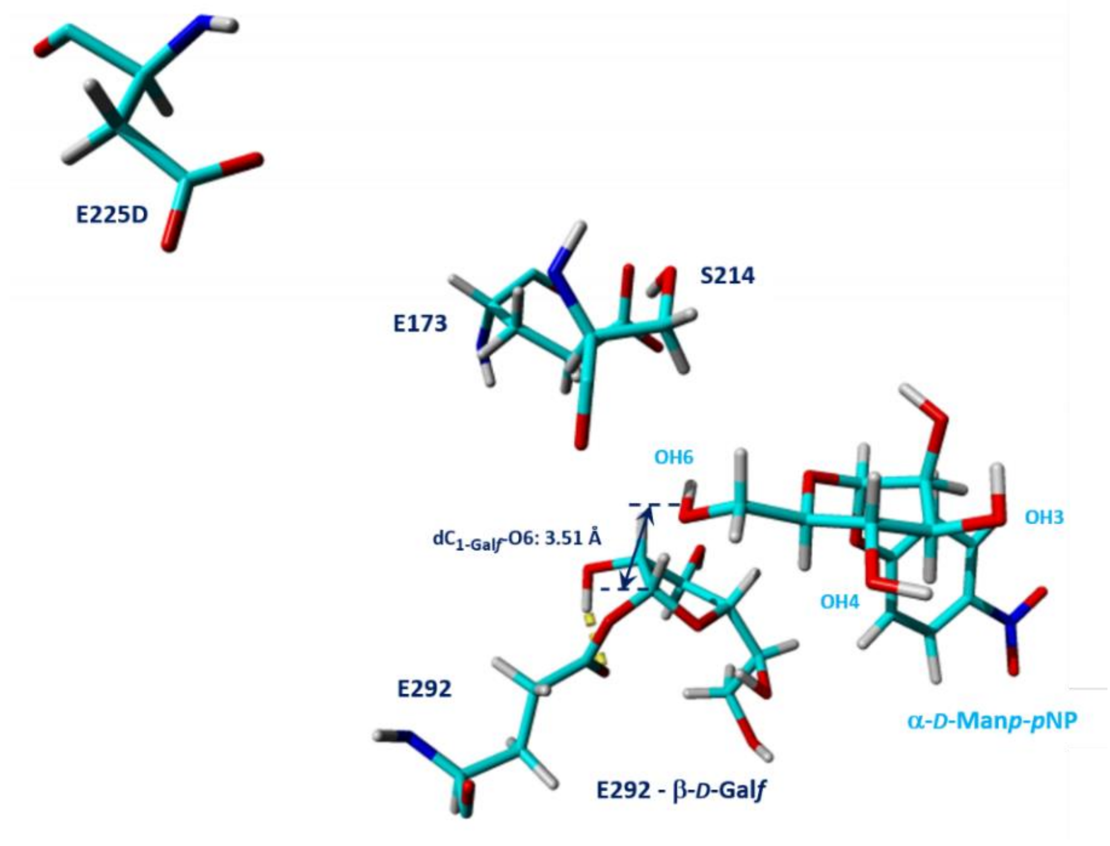

Figure S3(D1, D2, D3): *Ct*Ara*f*51 E225D-Gal*f* intermediate interacting with Man*p*-*p*NP after MD starting from the different (1→X)-oriented Man*p*-*p*NP (X = 3, 4, 6).

E1 *Ct*Ara*f*51 D327N / (1→3)

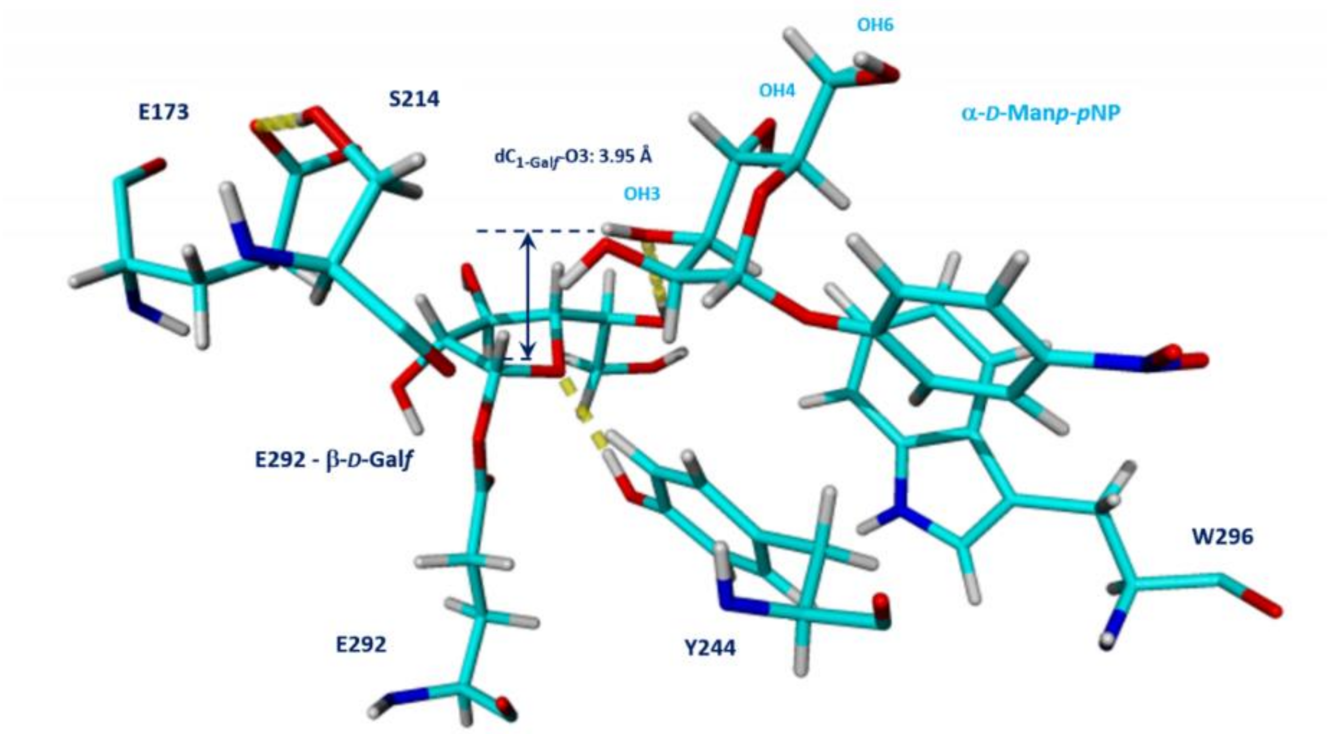

E2 *Ct*Ara*f*51 D327N / (1→4)

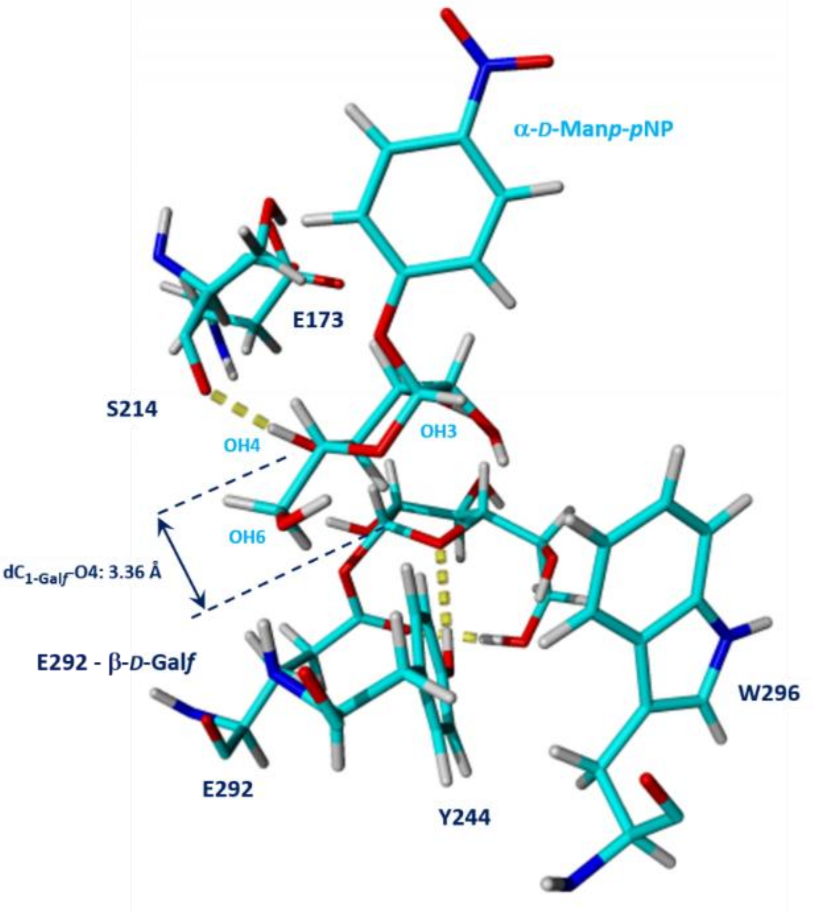

../...

E3 *Ct*Ara*f*51 D327N / (1→6)

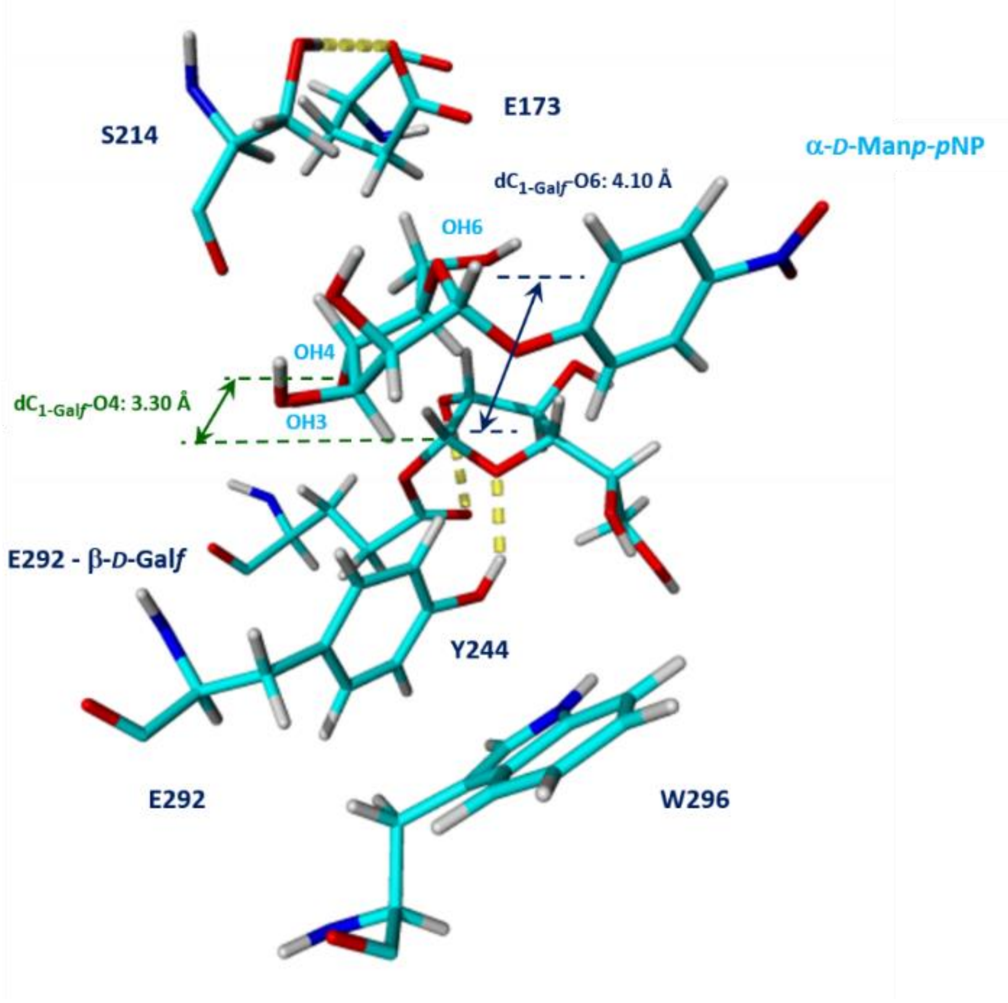

Figure S3(E1, E2, E3): *Ct*Ara*f*51 D327N-Gal*f* intermediate interacting with Man*p*-*p*NP after MD starting from the different (1→X)-oriented Man*p*-*p*NP (X = 3, 4, 6).

../...

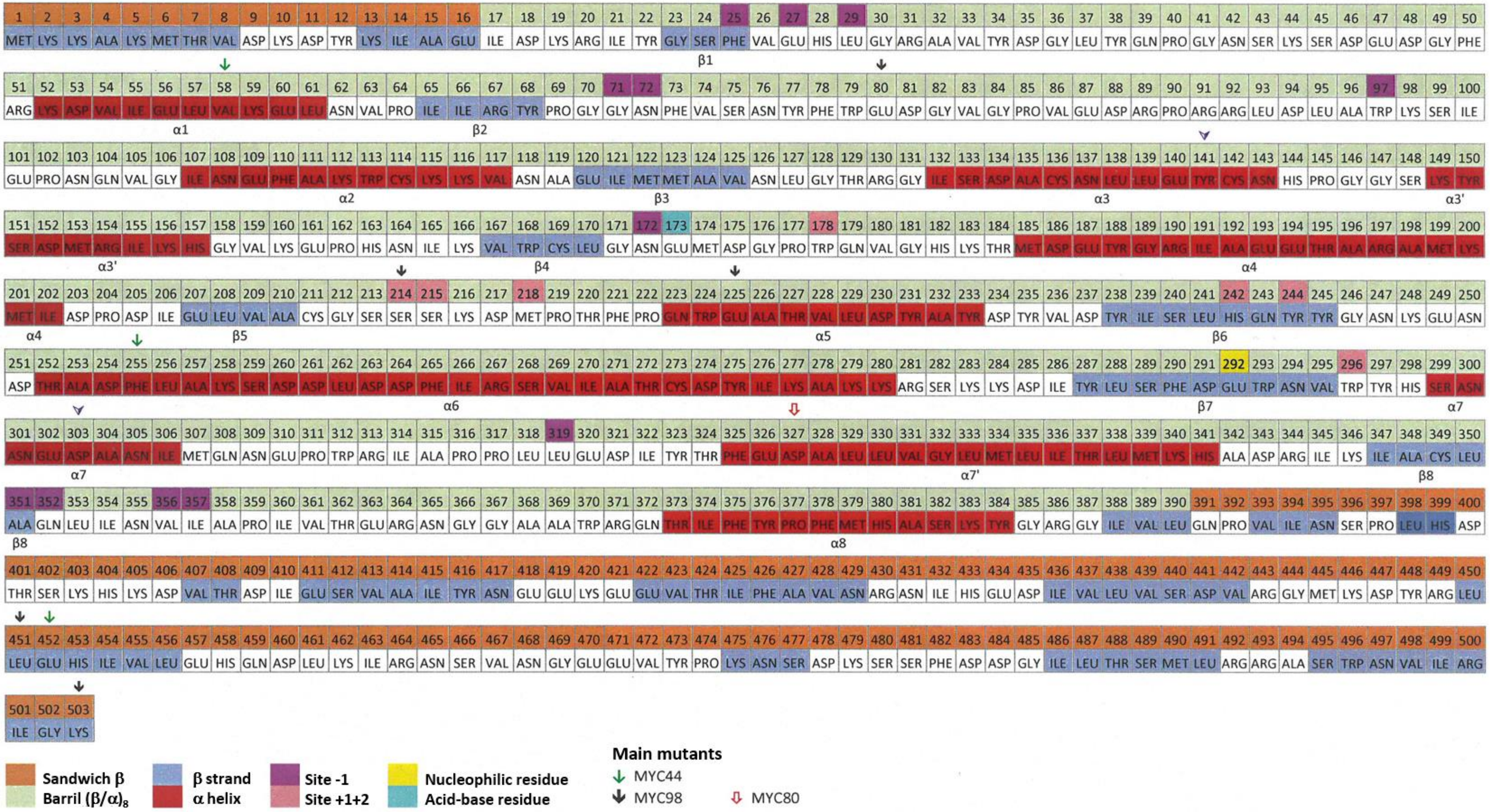

Figure S4: Secondary structure of *Ct*Ara*f*51 wild type and selected mutants.

The figures S5 were divided in three parts (A, B, C) corresponding to each initial (1→X)-orientations from the acceptor Man*p*-*p*NP (X = 3, 4, 6) in order to compare *Ct*Ara*f*51 WT and *Ct*Ara*f*51 S214T. The scale of B factors ranges from a value 0 corresponding to blue (cool, frozen) until 70 with yellow (hot, flexible).

A1 *Ct*Ara*f*51 Wild type / (1→3)

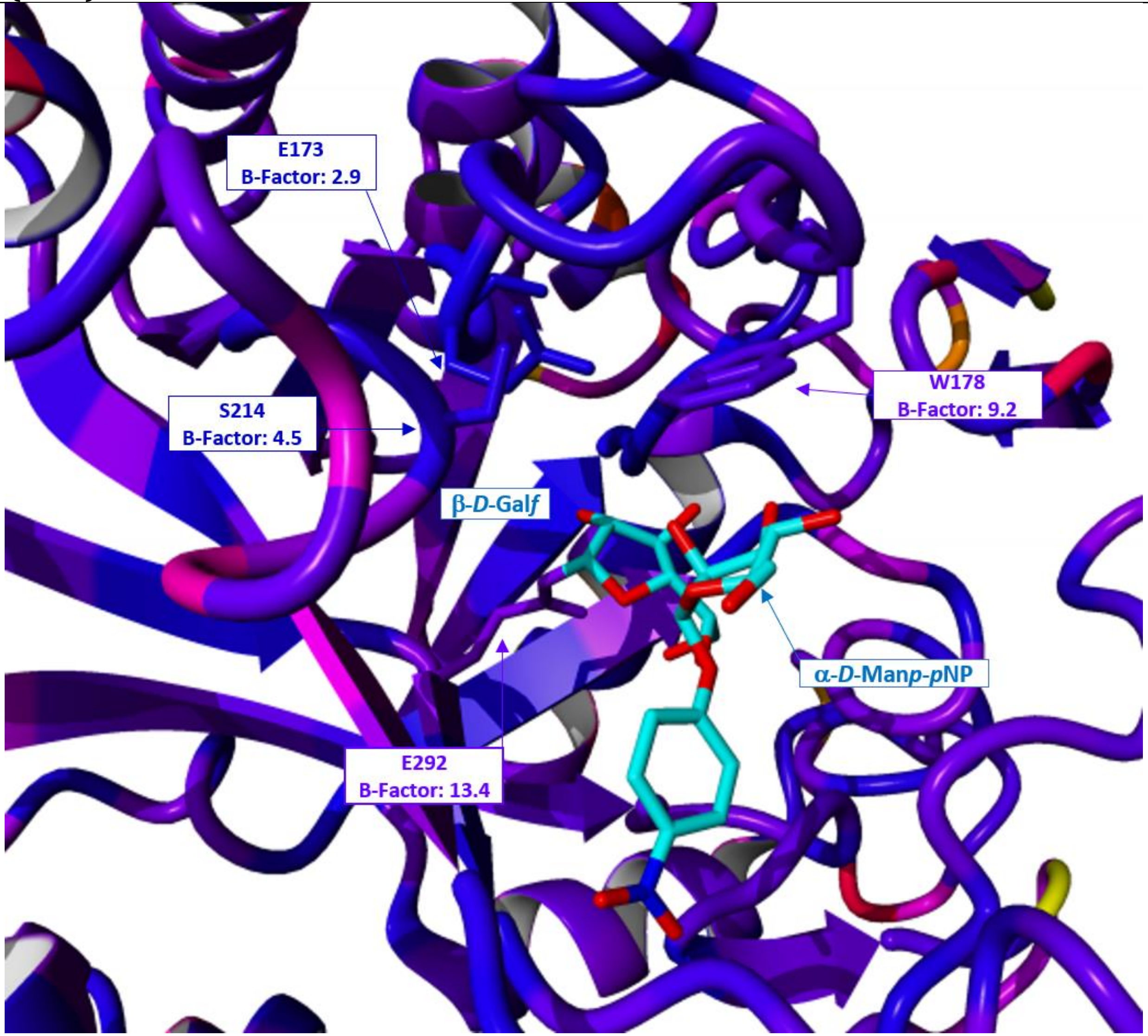

../...

A2 *Ct*Ara*f*51 S214T / (1→3)

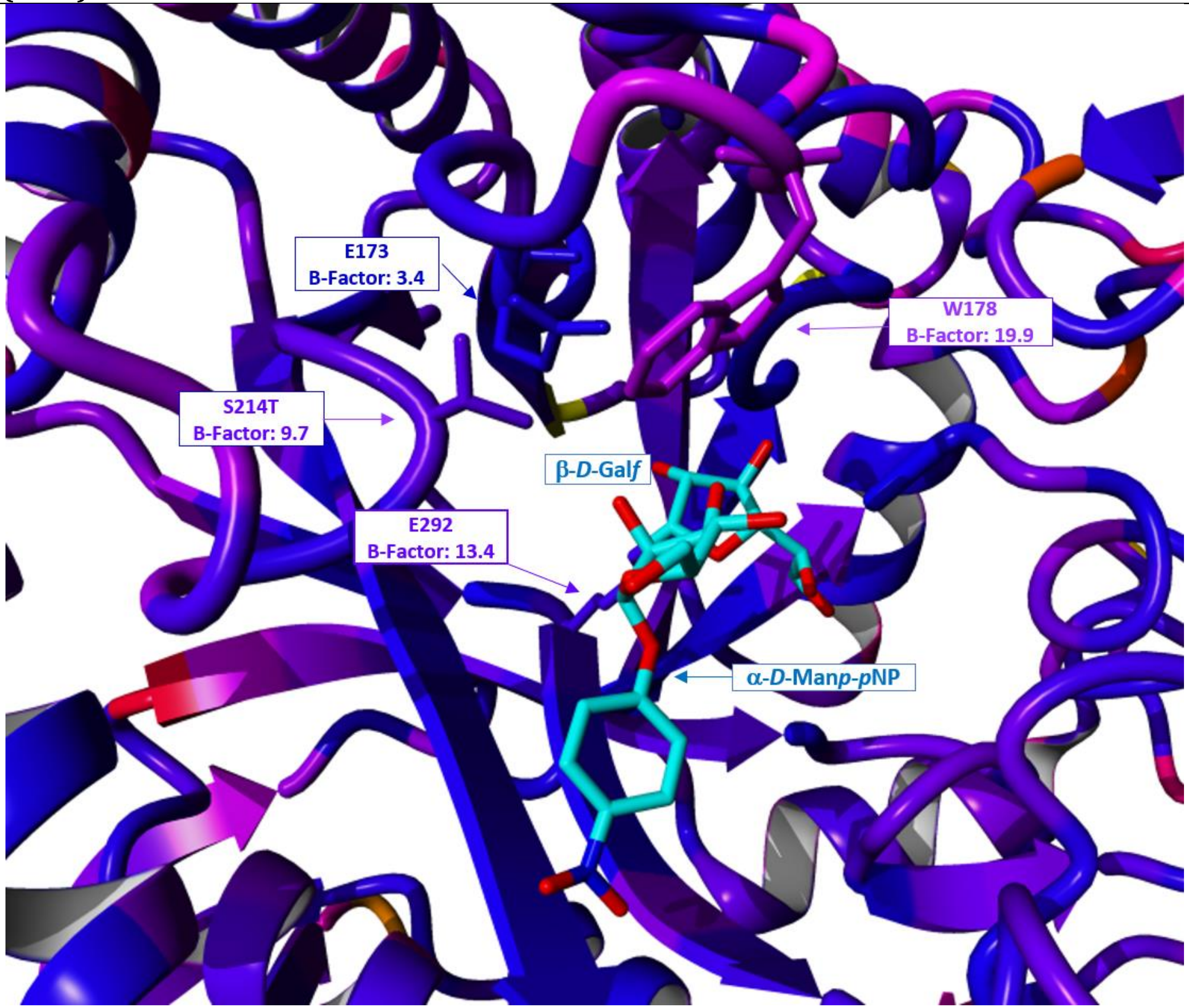

Figure S5(A1, A2): B-Factor maps per residue on MD complexes for *Ct*Ara*f*51WT-Gal*f* and *Ct*Ara*f*51 S214T-Gal*f* intermediates interacting with Man*p*-*p*NP according to its initial (1→3)-orientation.

B1 *Ct*Ara*f*51 Wild type / (1→4)

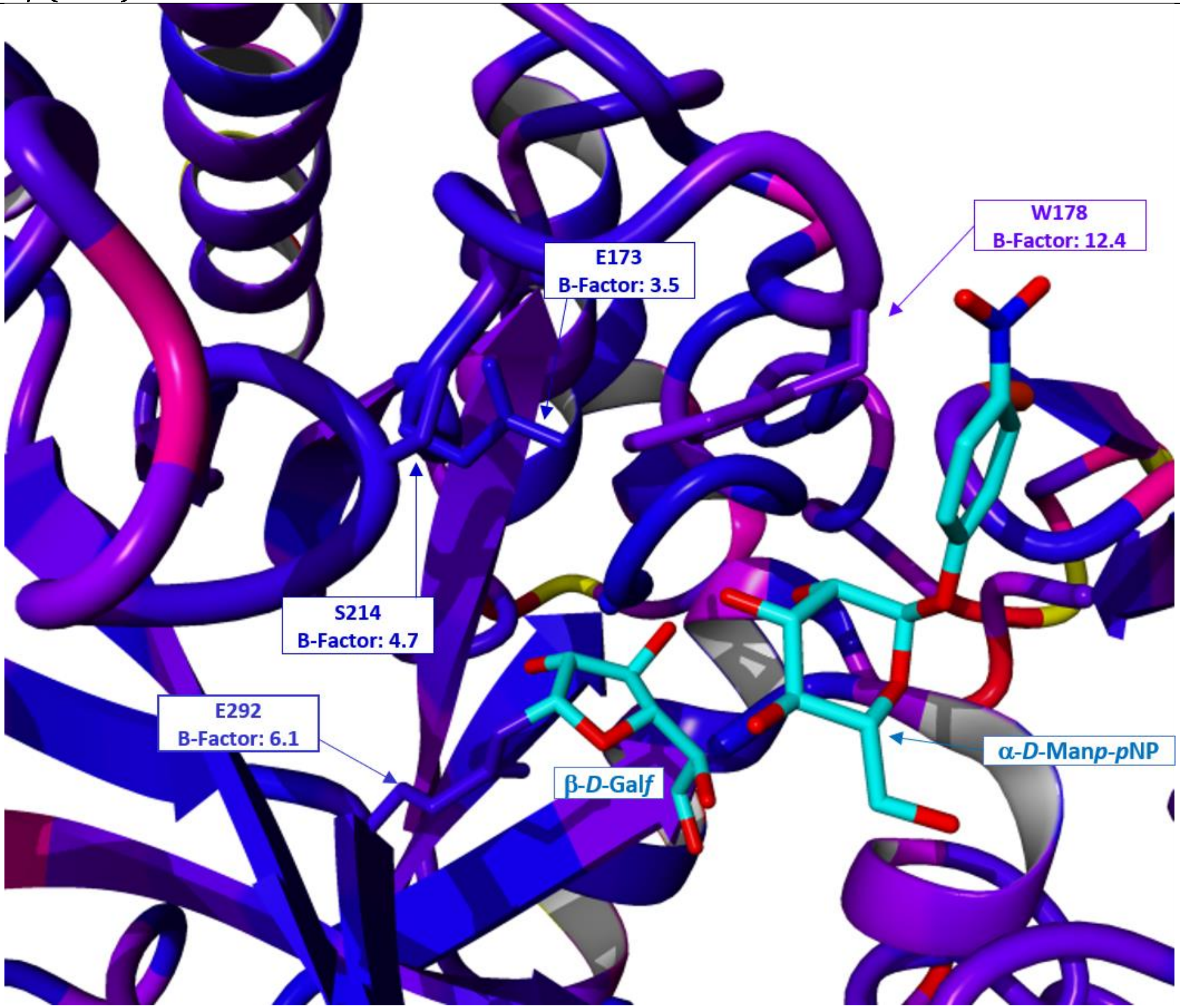

../...

B2 *Ct*Ara*f*51 S214T / (1→4)

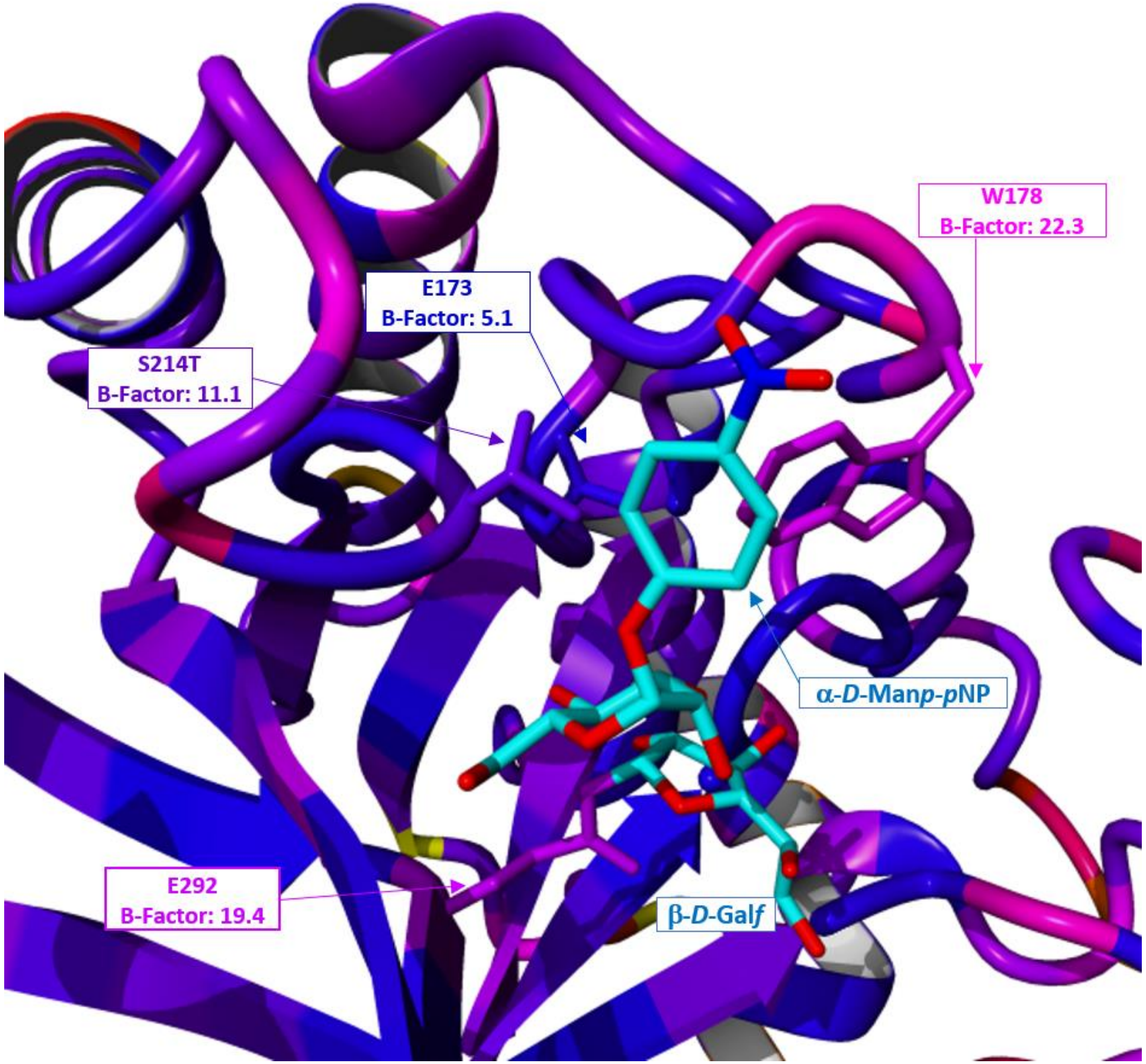

Figure S5(B1, B2): B-Factor maps per residue on MD complexes for *Ct*Ara*f*51WT-Gal*f* and *Ct*Ara*f*51 S214T-Gal*f* intermediates interacting with Man*p*-*p*NP according to its initial (1→4)-orientation.

C1 *Ct*Ara*f*51 Wild type / (1→6)

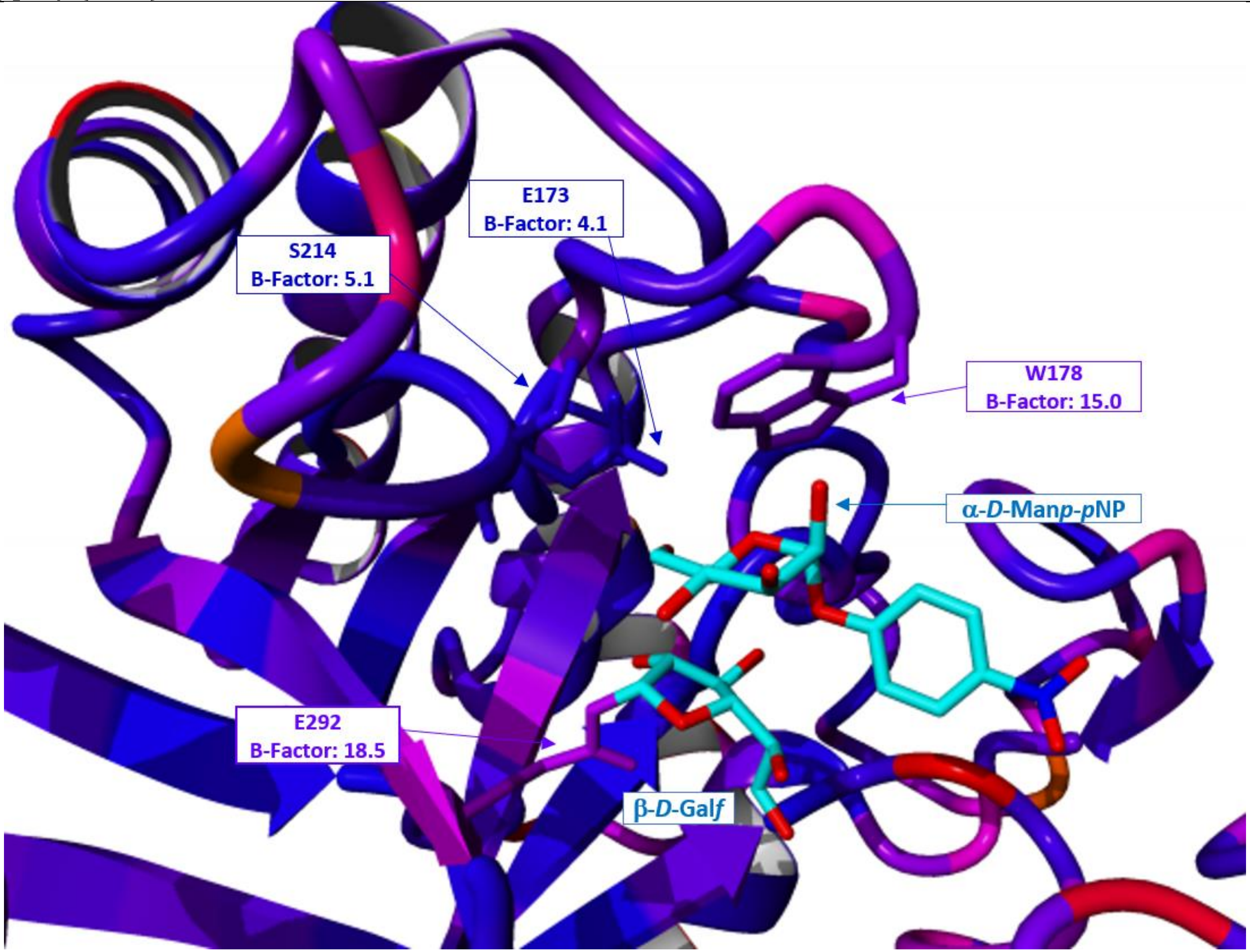

../…

C2 *Ct*Ara*f*51 S214T / (1→6)

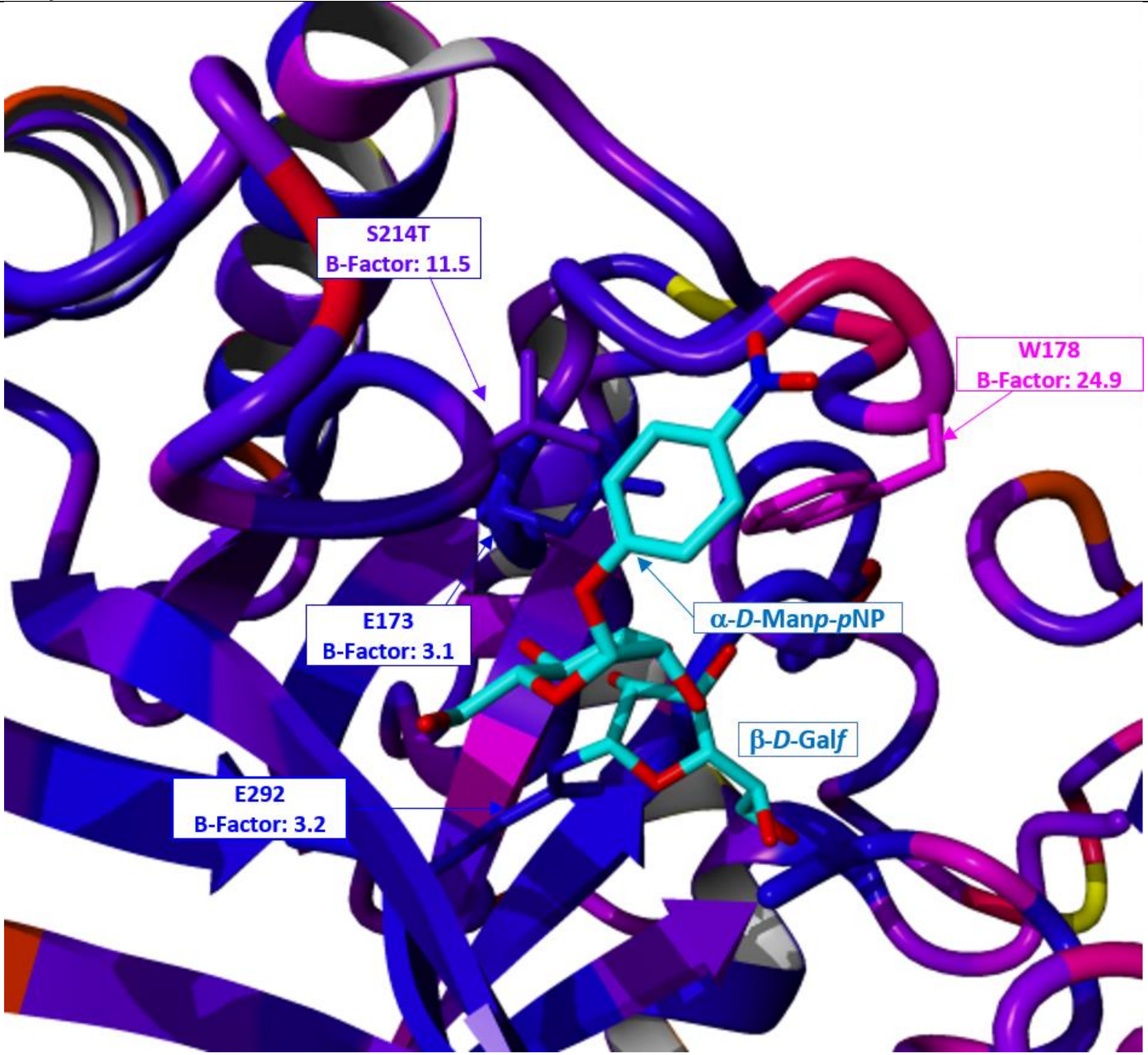

Figure S5(C1, C2): B-Factor maps per residue on MD complexes for *Ct*Ara*f*51WT - Gal*f* and *Ct*Ara*f*51 S214T-Gal*f* intermediates interacting with Man*p*-*p*NP according to its initial (1→6)-orientation.

As figures S5, the figures S6 were divided in same three parts (A, B, C) for each initial (1→X)-orientation (X = 3, 4, 6) in order to compare *Ct*Ara*f*51 WT and *Ct*Ara*f*51 D327N and present the same colorization according to the scale of B-factors.

A1 *Ct*Ara*f*51 Wild type / (1→3)

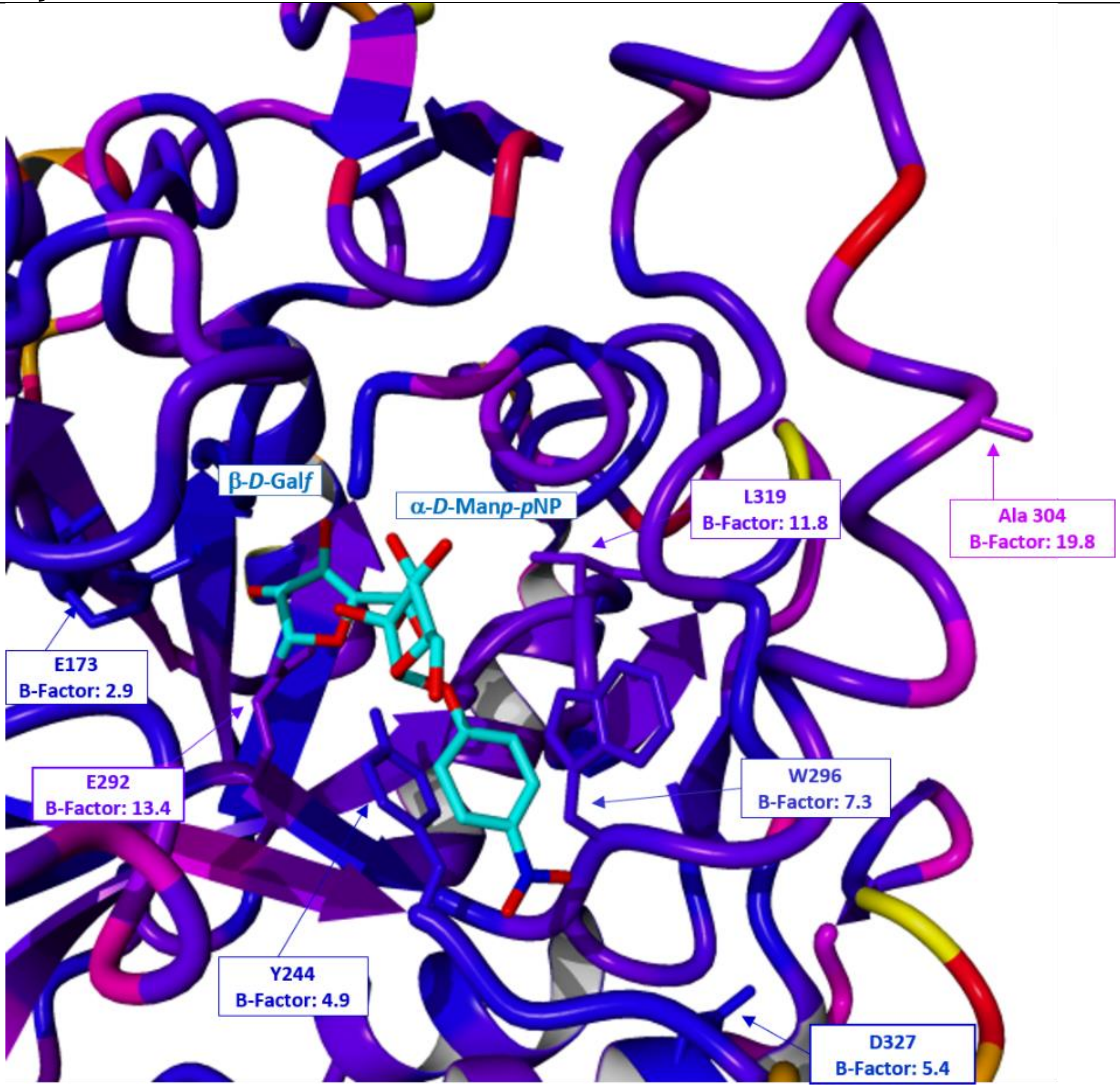

../…

A2 *Ct*Ara*f*51 D327N / (1→3)

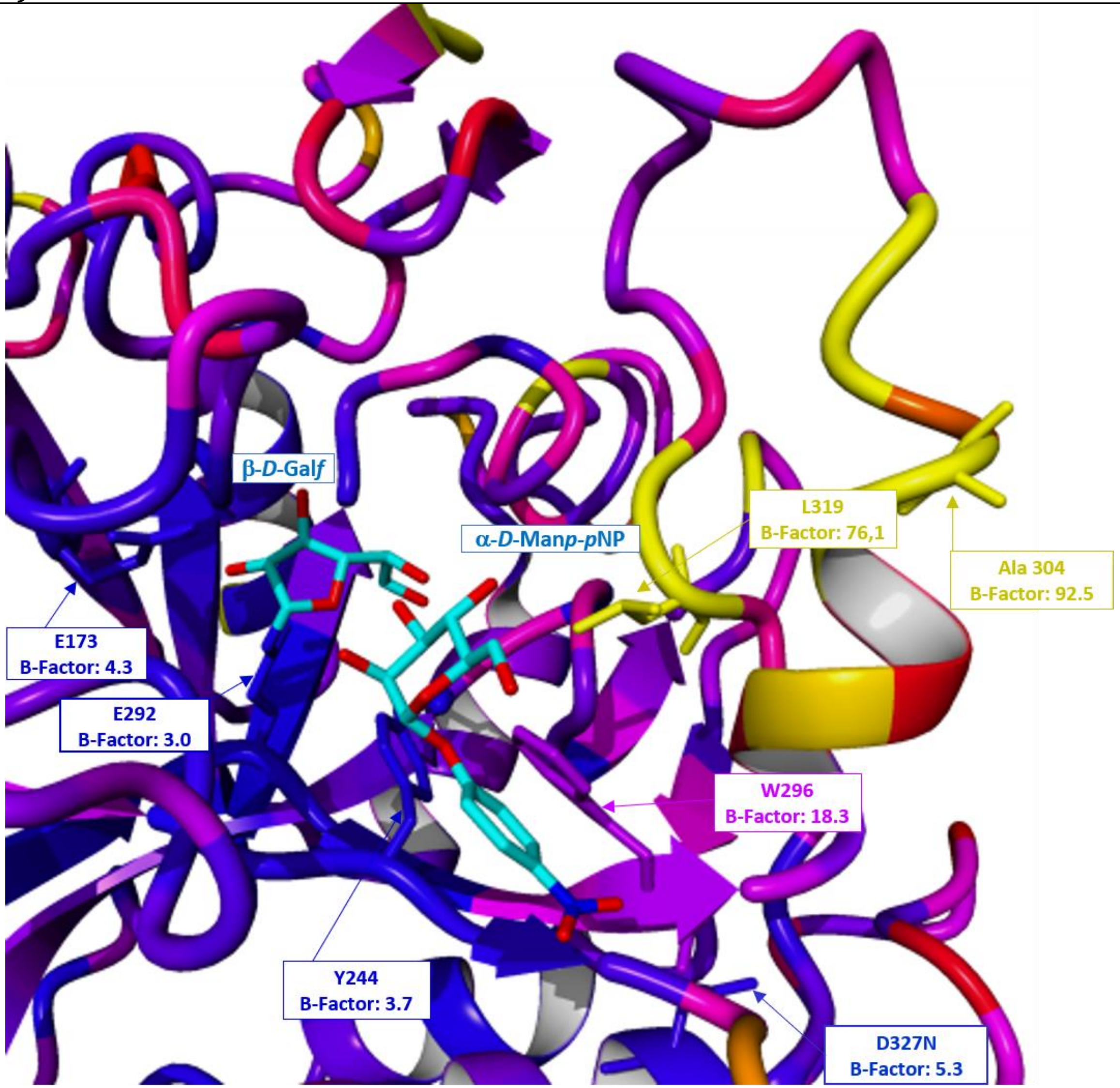

Figure S6(A1, A2): B-Factor maps per residue on MD for *Ct*Ara*f*51 WT and *CtAraf51* D327N-glycosyl-enzyme interacting with Man*p*-*p*NP according to its initial (1→3)-orientation.

B1 *Ct*Ara*f*51 Wild type / (1→4)

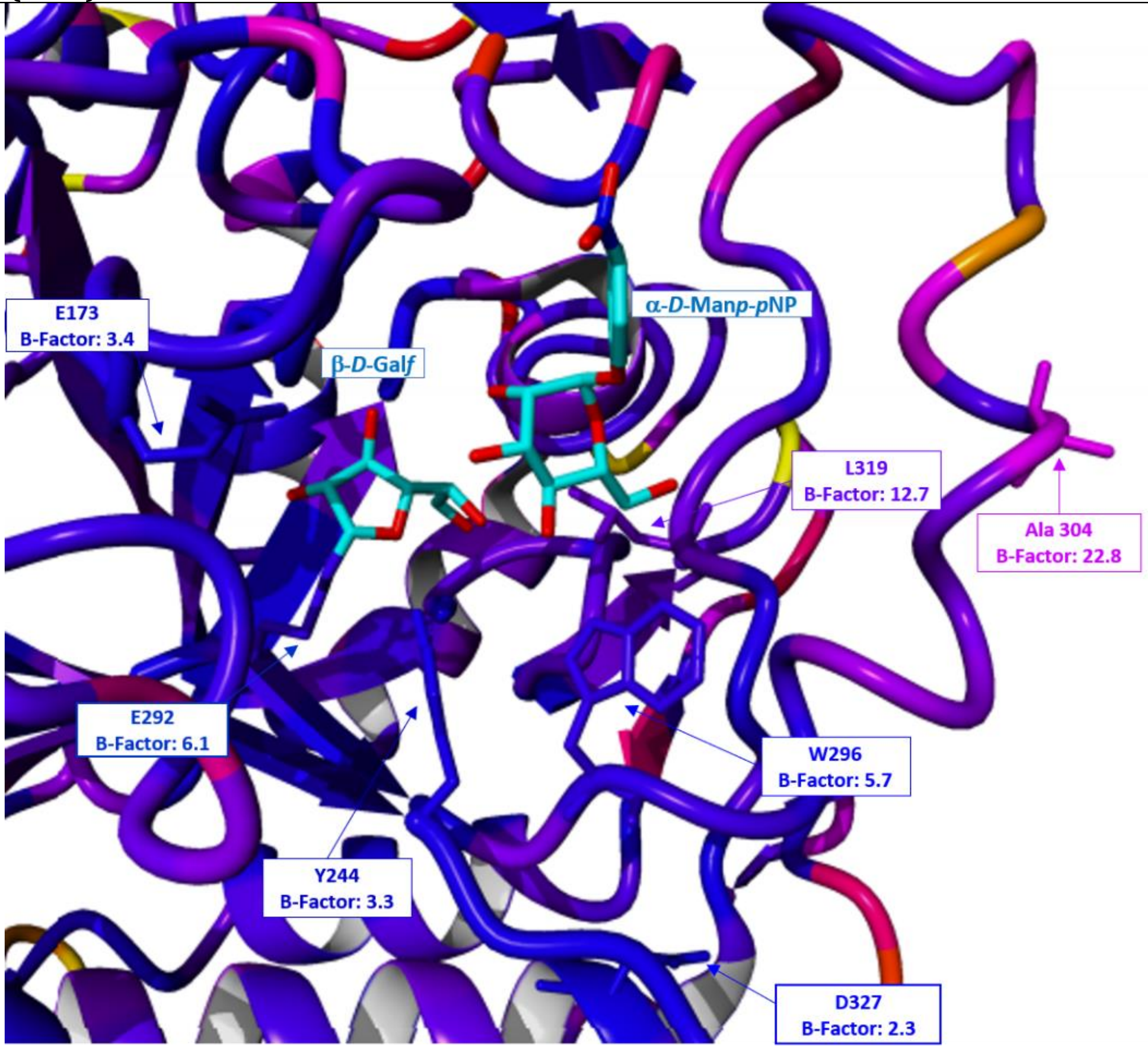

../...

B2 *Ct*Ara*f*51 D327N / (1→4)

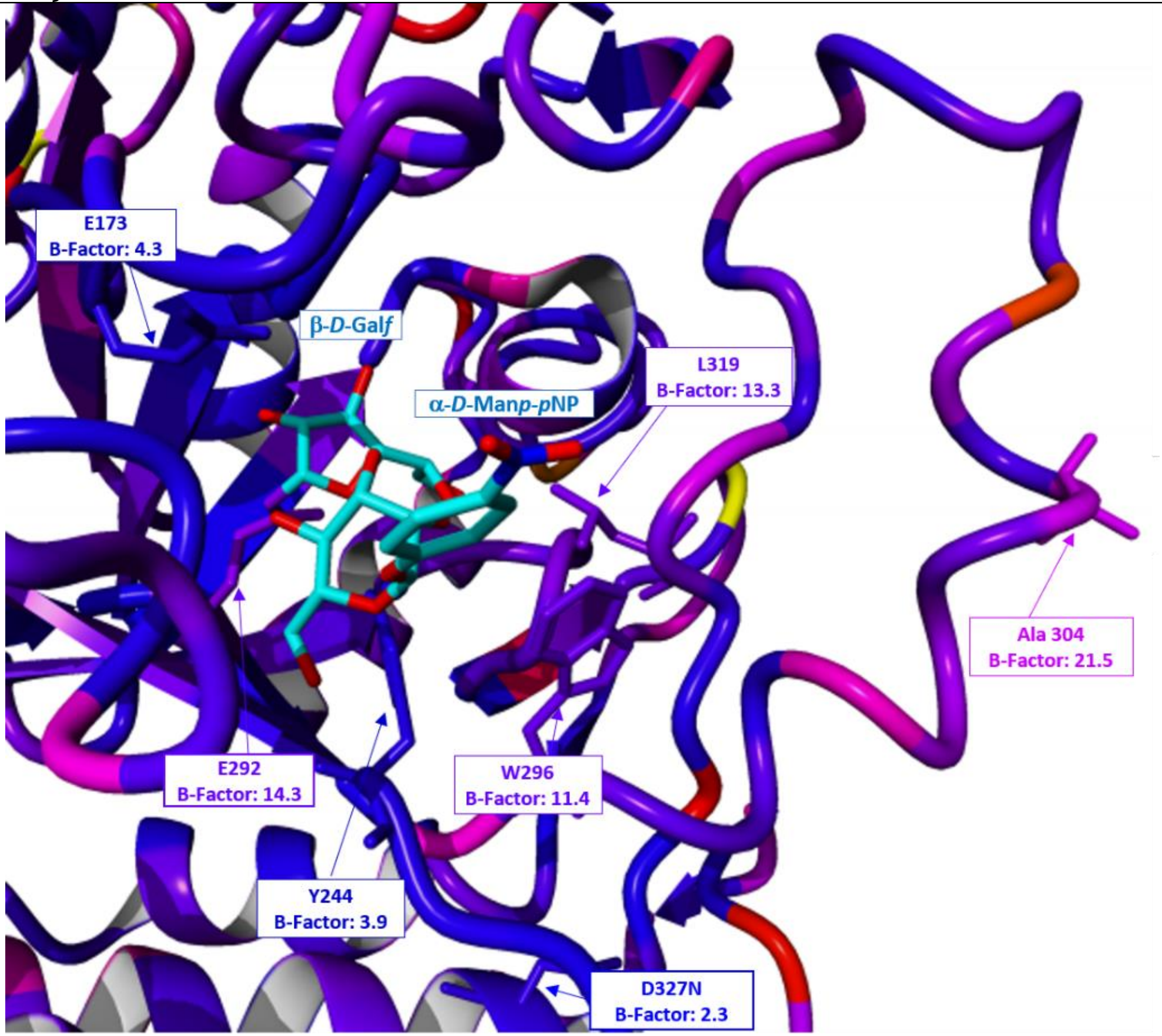

Figure S6(B1, B2): B-Factor maps per residue on MD for *Ct*Ara*f*51 WT and *CtAraf51* D327N-glycosyl-enzyme interacting with Man*p*-*p*NP according to its initial (1→4)-orientation.

../...

C1 *Ct*Ara*f*51 Wild type / (1→6)

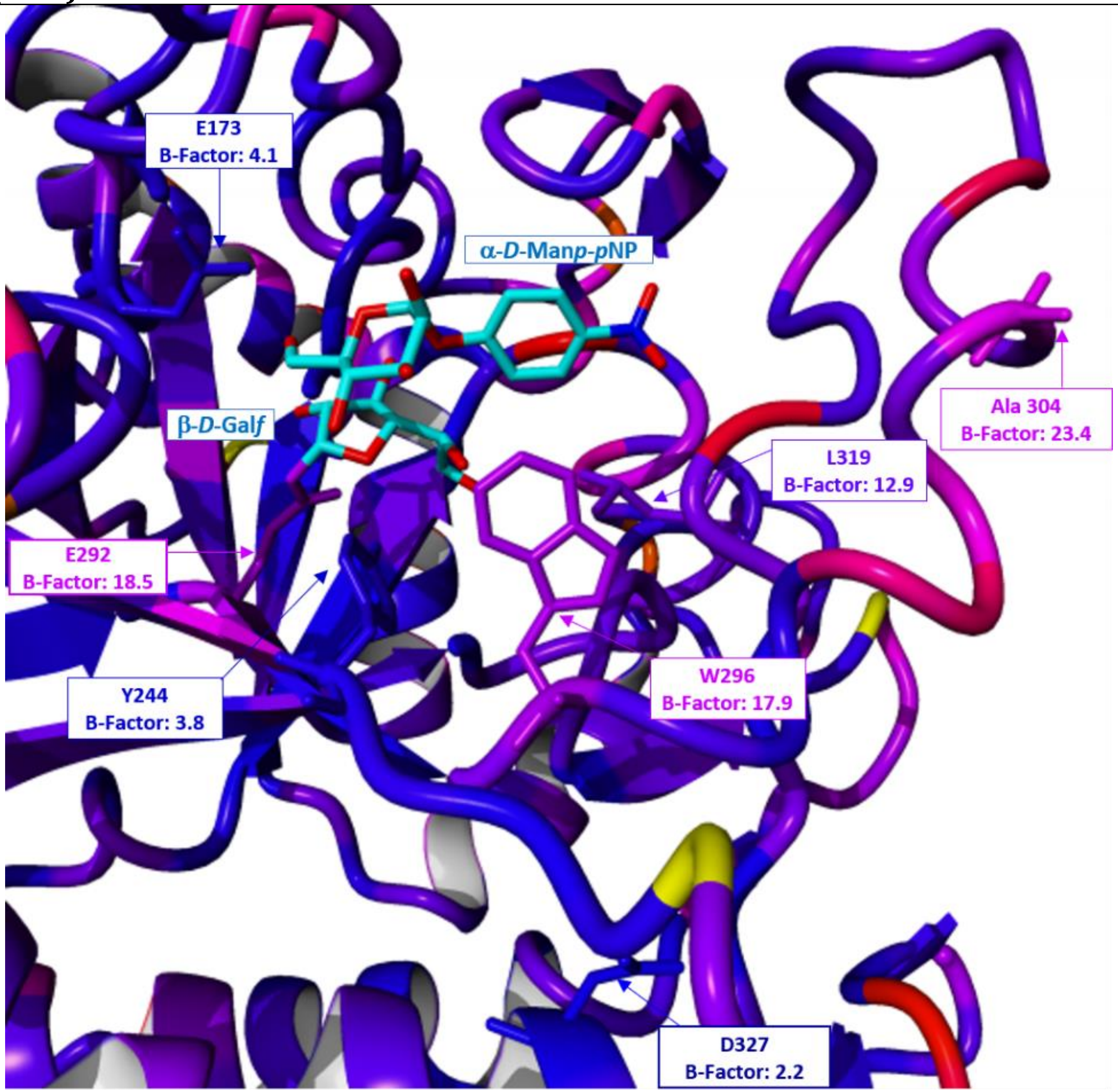

../…

C2 *Ct*Ara*f*51 D327N / (1→6)

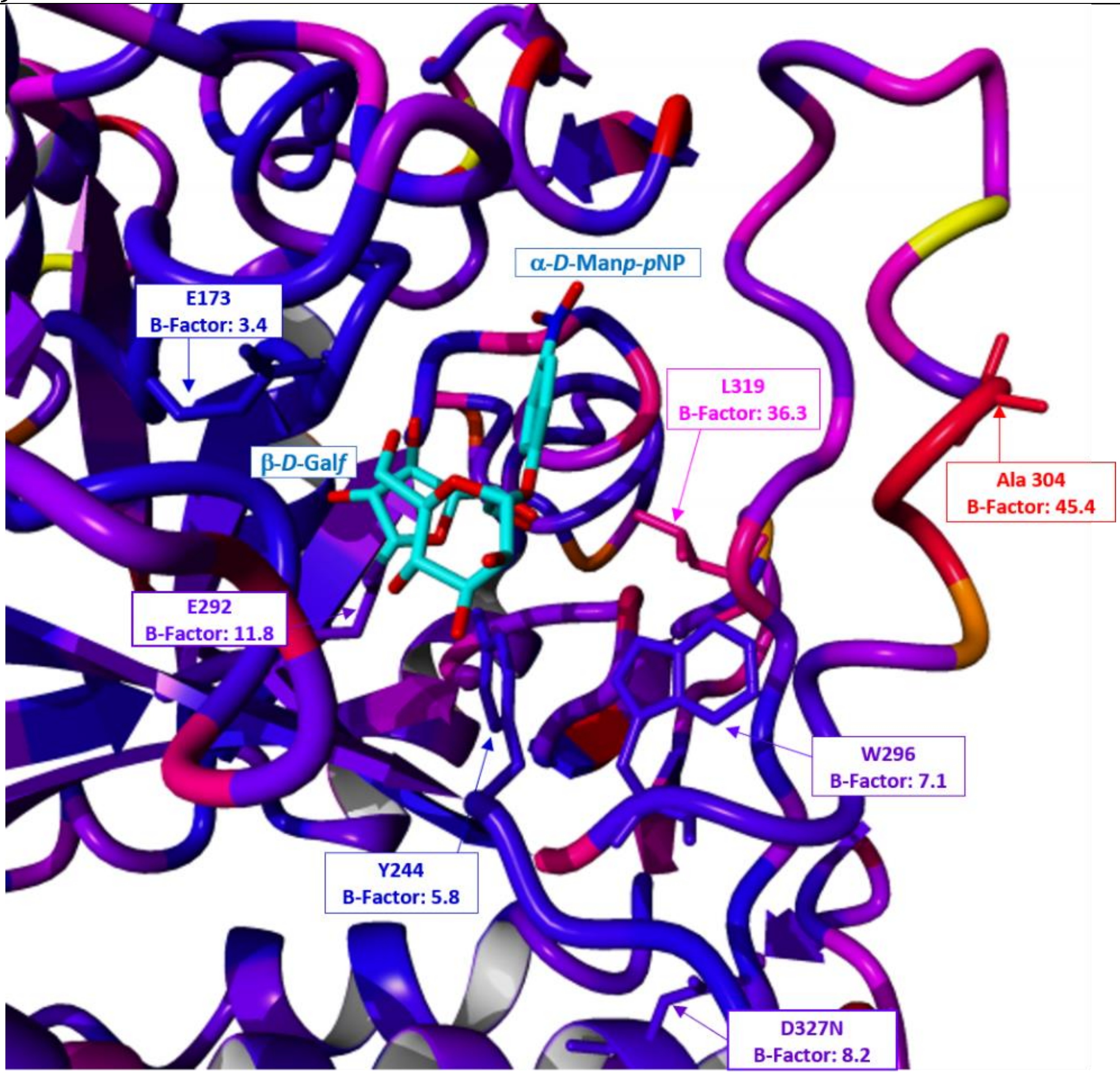

Figure S6(C1, C2): B-Factor maps per residue on MD for *Ct*Ara*f*51 WT and *CtAraf51* D327N-glycosyl-enzyme interacting with Man*p*-*p*NP according to its initial (1→6)-orientation.

SE1: Synthesis of the pre-screen donor substrate 5-bromo-indolyl β-D-galactofuranoside (5-BI-Gal*f*) (adapted from Berlin et al.[11]).

Perbenzoylated galactofuranosyl bromide and 1-acetyl-5-bromo-indoxyl-3-ol were obtained according to reported procedures.[11,12] Perbenzoylated galactofuranosyl bromide (920 mg, 1.4 mmol), 1-acetyl-5-bromo-indoxyl-3-ol (355 mg, 1.4 mmol) and powdered 4-Å molecular sieves were placed under nitrogen gas. Compounds were dissolved in 40 mL of dry dichloromethane. The mixture was cooled at 0 °C and kept protected from light. Silver triflate (362 mg, 1.4 mmol) was added quickly in one portion and the solution was stirred at room temperature during 1 h 30. The solution was filtered through a plug of celite. The filtrate was concentrated and purified by silica gel chromatography (cyclohexane/EtOAc, 8:2) to afford 823 mg of 1-*N*-acetyl-5-bromo-3-indolyl 2,3,5,6-tetra-*O*-benzoyl-β-D-galactofuranoside in 71 % yield. ^{1}H NMR (400 MHz, $CDCl_3$) δ (ppm): 8.28-7.24 (m, 24 H, H_{arom}), 6.11-6.07 (m, 1H, H-5), 5.91 (s, 1H, H-1), 5.89 (d, $J_{2,3}$ = 1.2 Hz, 1H, H-2), 5.79 (dd, 1H, $J_{3,4}$ = 4.8 Hz, H-3), 4.85 (dd, 1H, $J_{5,6a}$ = 5.2 Hz, $J_{6a,6b}$ = 11.6 Hz, H-6a), 4.87-4.82 (m, 1H, H-4), 4.70 (dd, 1H, $J_{5,6b}$ = 6.8 Hz, H-6b). 2.44 (s, 3H, CH_3). ^{13}C NMR (100 MHz, $CDCl_3$) δ (ppm): 168.3, 165.9, 165.6, 165.5, 165.4 (C=O), 139.2, 133.7, 133.4, 133.1, 132.2, 129.9, 129.4, 129.2, 129.1, 129.0, 128.6, 128.5, 128.4, 128.3, 120.5, 108.0 ($C_{indolyl}$, C_6H_5), 105.0 (C-1), 82.8 (C-4), 81.6 (C-2), 76.6 (C-3), 70.1 (C-5), 62.7 (C-6), 23.5 (CH_3).

1-*N*-acetyl-5-bromo-3-indolyl 2,3,5,6-tetra-*O*-benzoyl-β-D-galactofuranoside (0.29 g, 0.35 mmol) was added in a 0.1 M solution of sodium methylate in methanol (0.1 equivalent). After stirring for 3 h at room temperature, the reaction mixture was neutralized by adding Amberlite IR-120 (H^+-form). The resin was filtered off and the solvent removed under reduced pressure. The resulting crude material was purified by column chromatography (CH_2Cl_2/MeOH, 9:1) to give the 5-bromo-indolyl β-D-galactofuranoside (5-BI-Gal*f*) as a slightly blue/green paste in a 90 % yield. ^{1}H NMR (400 MHz, CD_3OD) δ (ppm): 7.60 (dd, 1H. $J_{4',6'}$ = 2.0 Hz, $J_{4',7'}$ = 0.8 Hz, H-4'), 7.17 (dd, 1H, $J_{7',6'}$ = 8.7 Hz, H-7'), 7.06 (dd, 1H, H-6'), 6.94 (s, 1H, H-2'), 5.20 (d, 1H, $J_{1,2}$ = 2.0 Hz, H-1), 4.19 (dd, 1H, $J_{2,3}$ = 4.0 Hz, H-2), 4.06-4.02 (m, 2H, H-3, H-4), 3.69-3.65 (m, 1H, H-5), 3.58 (dd, 1H, $J_{6a,5}$ = 5.5 Hz, $J_{6a,6b}$ = 11.0 Hz, H-6a), 3.54 (dd, 1H, $J_{6b,5}$ = 7.3 Hz, H-6b). ^{13}C NMR (100 MHz, CD_3OD) δ (ppm): 135.5, 133.7, 126.5 ($C\text{-}6_{indolyl}$), 123.1, 121.1 ($C\text{-}4_{indolyl}$), 115.8 ($C\text{-}7_{indolyl}$), 115.2, 113.4 ($C\text{-}2_{indolyl}$), 110.8 (C-1), 85.0 (C-4), 82.4 (C-2), 78.0 (C-3), 72.0 (C-5), 64.4 (C-6). HRMS (ESI^+): $[M + Na]^+$ ($C_{14}H_{16}NO_6BrNa$) m/z calculated: 396.0058; m/z found: 396.0057.

G(1→X)M disaccharides characterizations.

p-nitrophenyl β-D-galactofuranosyl-(1→6)-α-D-mannopyranoside (G(1→6)M):

^{1}H NMR (400 MHz, CD_3OD): δ (ppm): 8.24 (d, 2H, J_m = 9.2Hz, H_m C_6H_4), 7.29 (d, 2H, J_o=9.2Hz, H_o C_6H_4), 5.61 (d, 1H, $J_{1,2}$ = 1.8 Hz, H-1), 4.93 (d, 1H, $J_{1',2'}$ = 1.3 Hz, H-1'), 4.04 (dd, 1H, $J_{2,3}$ = 3.3 Hz, H-2), 3.95 (dd, 1H, $J_{3',4'}$ = 6.0 Hz, H-3'), 3.94 (d, 1H, $J_{6a,5}$ = 8.0 Hz, H-6a), 3.88 (dd, 1H, $J_{3,4}$ = 9.1 Hz, H-3), 3.87 (dd, 1H, $J_{2',3'}$ = 3.7 Hz, H-2'), 3.88-3.84 (m, 1H, H-4'), 3.73 (dd, 1H, $J_{4,5}$ = 8.6 Hz, H-4), 3.69-3.65 (m, 1H, H-5'), 3.66 (d, 1H, $J_{6b,5}$ = 8.0 Hz, H-6b), 3.65-3.61 (m, 1H, H-5), 3.57 (d, 2H, $J_{6',5'}$ = 6.2 Hz, H-6'). ^{13}C NMR (100 MHz, CD_3OD): δ (ppm): 126.7 (C_m C_6H_4), 118.0 (C_o C_6H_4), 109.8 (C-1'), 100.2 (C-1), 84.8 (C-4'), 83.0 (C-2'), 79.1 (C-3'), 74.9 (C-5), 72.5 (C-5'), 72.2 (C-3), 71.5 (C-2), 68.5 (C-4), 68.0 (C-6), 64.5 (C-6'). MS (ESI$^-$): $[M+HCOO]^-$ $C_{19}H_{26}NO_{15}$; m/z calculated: 508.13; m/z found: 508.05.

p-nitrophenyl β-D-galactofuranosyl-(1→4)-α-D-mannopyranoside (G(1→4)M):

^{1}H NMR (400 MHz, CD_3OD): δ (ppm): 8.23 (d, 2H, J_m = 9.2Hz, H_m C_6H_4), 7.29 (d, 2H, J_o = 9.2Hz, H_o C_6H_4), 5.66 (d, 1H, $J_{1,2}$ = 1.8 Hz, H-1), 5.03 (d, 1H, $J_{1',2'}$ = 2.0 Hz, H-1'), 4.13 (dd, 1H, $J_{4',3'}$ = 3.3 Hz, $J_{4',5'}$ = 6.4 Hz, H-4'), 4.10 (dd, 1H, $J_{2,3}$ = 3.1 Hz, H-2), 4.04 (dd, 1H, $J_{3',2'}$ = 6.4 Hz, H-3'), 4.01 (dd, 1H, $J_{3,4}$ = 9.4 Hz, H-3), 3.96 (dd, 1H, $J_{2',3'}$ = 4.4 Hz, H-2'), 3.95 (app. t, 1H, $J_{4,5}$ = 9.2 Hz, H-4), 3.74-3.70 (m, 3H, H-5', H-6), 3.65-3.61 (m, 2H, H-6'), 3.61-3.57 (m, 1H, H-5'). ^{13}C NMR (100 MHz, CD_3OD): δ (ppm): 126.7 (C_m C_6H_4), 117.7 (C_o C_6H_4), 109.8 (C-1'), 99.8 (C-1), 84.5 (C-4'), 83.0 (C-2'), 78.1 (C-3'), 75.5 (C-4), 74.6 (C-5), 72.2 (C-5'), 71.3 (C-2), 70.9 (C-3), 64.3 (C-6'), 61.8 (C-6). MS (ESI$^-$): $[M+HCOO]^-$ $C_{19}H_{26}NO_{15}$; m/z calculated: 508.13; m/z found: 508.05.

p-nitrophenyl β-D-galactofuranosyl-(1→3)-α-D-mannopyranoside (G(1→3)M):

^{1}H NMR (400 MHz, CD_3OD): δ (ppm): 8.23 (d, 2H, J_m = 9.2Hz, H_m C_6H_4), 7.29 (d, 2H, J_o = 9.2Hz, H_o C_6H_4), 5.70 (d, 1H, $J_{1,2}$ = 1.9 Hz, H-1), 5.17 (d, 1H, $J_{1',2'}$ = 1.0 Hz, H-1'), 4.24 (dd, 1H, $J_{2,3}$ = 3.2 Hz, H-2), 4.14 (dd, 1H, $J_{4',3'}$ = 3.3 Hz, $J_{4',5'}$ = 5.3 Hz, H-4'), 4.10-4.05 (m, 3H, H-2', H-3', H-3), 3.85 (app. t, 1H, $J_{4,5}$ = 9.7 Hz, H-4), 3.79-3.61 (m, 5H, H-5', H-6, H-6'), 3.57-3.53 (m, 1H, H5). ^{13}C NMR (100 MHz, CD_3OD): δ (ppm): 162.5 (C_{ipso} C_6H_4), 143.8 (C_p C_6H_4), 126.7 (C_m C_6H_4), 117.7 (C_o C_6H_4), 106.6 (C-1'), 100.0 (C-1), 85.6 (C-4'), 82.7 (C-2'), 79.1 (C-3'), 76.8 (C-3), 76.0 (C-5), 72.6 (C-5'), 68.2 (C-2), 66.4 (C-4), 64.3 (C-6'), 62.6 (C-6). MS (ESI$^-$): $[M+HCOO]^-$ $C_{19}H_{26}NO_{15}$; m/z calculated: 508.13; m/z found: 508.05.

p-nitrophenyl β-D-galactofuranosyl-(1→2)-α-D-mannopyranoside (G(1→2)M):

^{1}H NMR (400 MHz, CD_3OD): δ (ppm): 8.23 (d, 2H, J_m = 9.2Hz, H_m C_6H_4), 7.29 (d, 2H, J_o = 9.2Hz, H_o C_6H_4), 5.84 (d, 1H, $J_{1,2}$ = 1.5 Hz, H-1), 5.17 (app . s, 1H, H-1'), 4.27 (dd, 1H, $J_{2,3}$ = 3.5 Hz, H-2), 4.21-4.17 (m, 1H, H-3'), 4.07-4.03 (m, 1H, H-2'), 3.93 (dd, 1H, $J_{3,4}$ = 9.5 Hz, H-3), 3.77-3.73 (m, 1H, H-4'), 3.70-3.57 (m, 6H, H-5', H-4, H-6', H-6), 3.53-3.49 (m, 1H, H-5)*. MS (ESI$^-$): $[M+HCOO]^-$ $C_{19}H_{26}NO_{15}$; m/z calculated: 508.13; m/z found: 508.05.

* (G(1→2)M) was obtained in mixture with others disaccharides at very low yield, ^{13}C NMR characterization remained incompleted.
